# Web of Power in the Struggle for Access Rights to Forest Resources: Comparative Case Studies in Conservation and Industrial Forests

**DOI:** 10.12688/f1000research.171466.2

**Published:** 2026-06-09

**Authors:** Rita Rahmawati, Rusliandy Rusliandy, Imam Edy Mulyono, Sri Harini, Neng Virly Apriliyani, Euis Salbiah, Irma Purnamasari, R. Akhmad Munjin, Faisal Tri Ramdani, Berry Sastrawan, Evi Satispi, Andriansyah Andriansyah, Novel Anak Lyndon

**Affiliations:** 1 Public Administration, Universitas Djuanda, Bogor, West Java, 16720, Indonesia; 2 Management, Universitas Djuanda, Bogor, West Java, 16720, Indonesia; 3 Public Administration, Universitas Muhammadiyah Jakarta, Jakarta, Special Capital Region of Jakarta, 15419, Indonesia; 4 Social, Environment and Development Sustainability Research Center, Universiti Kebangsaan Malaysia Faculty of Social Sciences and Humanities, Bangi, Selangor, 43600, Malaysia

**Keywords:** Conflict and Contestation, Customary Rights, Inclusive natural resource governance, Indigenous People, Sociology of Governance.

## Abstract

The web of power in the struggle for forest resources arose as a response to forest resource conflicts between the state and local communities. In Foucault’s concept, power was not held solely by the state but existed in social relations involving various parties. This study aimed to map the web of power in the struggle for access rights to forest resources. It used a qualitative approach and a comparative case study method. The informants for this study were local governments, forest managers, local communities, and NGOs. Ten informants were selected purposively in each location. Data collection techniques were observation, in-depth interviews, and literature studies. Data analysis was carried out using thematic analysis. The study results showed that in Mount *Halimun Salak* National Park (TNGHS), conflict occurred due to the policy of expanding the national park, resulting in the loss of local community access to the forest area. Meanwhile, in *Sungai Utik,* the conflict was triggered by a state policy that issued a business permit to use forest products (IUPHHK) in the customary forest area. Although the IUPHHK has never been revoked, the power relations have shown a more egalitarian pattern based on the state’s recognition and the community’s solidarity. The findings indicated that contestation over access rights to forest resources in two different forests had resulted in two distinct webs of power models. Power relations were not static, but constantly changed depending on the configuration of local communities’ political power, legal support, advocacy networks, and social capital.

## Introduction

Power relations are the dynamics between political elites and local communities, shaped by their struggle to control forest resources. These power relations define the balance of power and serve as a community strategy to mobilize other stakeholders in their quest for power in settling disputes over forest access rights. These disputes, which evolve into meaningful conflicts between the involved parties, ^1^ have made access to natural resources, particularly forests, a pivotal issue in the power dynamics between the state, political elites, and local communities in Indonesia. Beyond their economic significance, forests are repositories of cultural, spiritual, and ecological values indispensable to local communities, especially indigenous ones. However, the unequal power relations in forest resource management often create tension and injustice.

Forest resource management is a technical and ecological challenge and a social arena fraught with complex power dynamics and management structures. From a sociological perspective, environmental management is seen as the product of interactions between various parties: the state, companies, local communities, NGOs, and others, each with its own interests, perspectives, and legitimacy methods. ^2^ In this context, power is relational and distributed, centralized in the state and emerging through narratives, regulatory practices, and even local knowledge that influence forest perspectives. ^3^ Hegemony sometimes occurs in power relations in forest management, where powerful actors exert hegemony through coercion or unintentional consent, while weaker groups resist through counter-hegemonic actions. ^4, 5^ Therefore, a comprehensive understanding of the web of power in the competition for forest access is crucial. It engages us in the struggle for ideas, the distribution of power, and forms of resistance and negotiation at both the local and global levels, and commits us to finding solutions.

Research on power relations was often linked to the reciprocal relationship between humans and natural resources. It examined how humans and nature were connected and how humans utilize nature. ^6^ Power relations significantly determined access to and utilization of resources. ^7^ In resource control, unequal power relations often led to conflict. The agrarian conflict in Aceh was caused by unequal land ownership between companies, farmers, and the government. ^8^ Several triggers for conflict exist, including injustice, restrictions on access to forest resources, and a lack of community involvement in forest management. Evictions from forests, acts of violence, and lawsuits were some of the events that contributed to the conflict pathway. ^9^ Conflicting policies and overlapping ownership arrangements, ^10^ also contributed to unequal power relations. Unfair utilization of natural resources led to conflict and tension in the struggle for natural resources. Tensions based on unequal power relations and hierarchical structures of actors had zero-sum impacts on humans and the physical environment. ^11^

History showed that in some regions, land and natural resource grabbing have resulted in the loss of rights for one party over another. Studies of land grabbing in China provided a glimpse into state-society relations in authoritarian versus democratic regimes. ^12^ In the case of forest resources, power relations became unequal due to differences in power and diverse interests in forest utilization and control. Under these conditions, forest conservation policies were negotiated, manipulated, and reformed in ways that differ significantly from those initially envisioned by policymakers. ^13^ In Nepal’s case of forest management, forest management plans were a prerequisite for obtaining forest management rights. Users accept the plans to gain access to forest resources, while for the forestry bureaucracy, the plans served as a tool to regain power and authority over the forests. ^14^ In Laos, land-use planning was crucial for strengthening land tenure security, intensifying agriculture, and conserving forest areas. ^10^ In Vietnam, restrictions on access rights to forest resources and a lack of alternative livelihoods have been a negative consequence of REDD+. ^15^ In Norway, rural unrest and center-periphery conflict could be caused by alienation, isolation, or estrangement from one’s community or society, leading to social and political unrest. ^16^ In Zimbabwe, the concept of local communities being excluded from access to resources in protected forest areas was also found. ^17^ Power is exercised through various mechanisms, processes, and social relationships, impacting local communities’ access to resources. ^17^ In India, land and natural resource grabbing are occurring to support ‘growth’. Marginalized groups and local communities were excluded from access to and control over resources. ^18^

Various cross-country studies have shown that forest management policies and REDD+ programs are not merely technical instruments. Instead, they are arenas of power relations that determine access to and distribution of resource benefits. A common thread across these cases is the duality of these policies. On one hand, they promote conservation and governance. On the other hand, they can strengthen the control of dominant actors and marginalize local communities, especially when power and participation are not inclusive. This situation is relevant to Indonesia. Despite a relatively strong REDD+ institutional design, regulatory barriers remain. ^19^ Unequal benefits persist for smallholders and indigenous communities due to weak political representation. ^20^ Challenges continue with funding, land boundaries, and community engagement. ^21^ Strengthening equitable access, participation, and distribution of power is key to ensuring REDD+ is not only ecologically effective but also socially just.

Recent international studies show that inclusive governance is key to environmentally sustainable and socially just development. Kato and Manchidi (2025) emphasized its role in responsible, equitable supply chain management in Africa. ^22^ Bibliometric research by Faheem et al. (2023) revealed a global trend toward international collaboration, green innovation, and inclusive natural resource governance. ^23^ Recent work also highlights the increasing importance of customary institutions and indigenous participation. For instance, integrating customary law into marine protected area management in Indonesia can improve conservation and local legitimacy. ^24^ Likewise, Swardhana and Jenvitchuwong (2023) argued that indigenous participation in land management is vital to equitable governance and protecting these communities under environmental and development pressures. ^25^

These developments show a broader shift in global environmental governance. Previously, models were hierarchical and top-down. Now, there is a move toward adaptive, participatory, and multi-level governance systems. These new systems value local ecological knowledge, customary institutions, and shared decision-making. In many countries, environmental conflicts arise not only from competition over resources but also from struggles for recognition, legitimacy, and authority in governance. Batiran et al. (2023) studied changes in Kelimutu National Park. ^26^ Their work showed how conservation policies can reshape indigenous institutions, creating new tensions between state agendas and local communities. The cases of Mount Halimun Salak National Park and the Sungai Utik customary forest contribute to global discussions about inclusive governance, political ecology, and indigenous environmental rights.

Recent developments in Indonesia’s forestry governance have been strongly influenced by the implementation of the Omnibus Law on Job Creation (Law No. 11 of 2020), later reinforced through Law No. 6 of 2023. The regulation introduced significant changes to environmental and forestry governance by simplifying licensing systems, centralizing authority, and accelerating investment mechanisms. Although the policy was intended to improve economic efficiency and national competitiveness, several studies have highlighted its implications for environmental governance, particularly concerning indigenous peoples’ rights, customary forest recognition, and conservation management that the implementation of the Omnibus Law has created challenges for forest decentralization in Indonesia by weakening political and administrative decentralization in forestry governance. ^27^^–^^29^ This condition can affect the recognition of customary forests and limit local communities’ participation in forest management processes. Furthermore, recent reviews of studies on the Omnibus Law have highlighted ongoing controversies surrounding its legal, social, and ecological implications, particularly regarding environmental protection and forest governance. ^30^ These studies suggest that regulatory simplification under the Omnibus Law may weaken participatory mechanisms and create overlapping authority between conservation policies, investment interests, and indigenous territorial claims. Similar concerns were also raised by Imamulhadi et al. (2025), who argued that the transformation of customary environmental law in Indonesia continues to face significant challenges due to the dominance of state-centered legal frameworks and the limited integration of customary governance systems into formal environmental policies. ^31^

Indigenous forest governance in Indonesia is fundamentally shaped by struggles over power and recognition. Studies on the Dayak Iban in West Kalimantan show that customary forest recognition is crucial for sustainable management and local protection. ^32^ The Dayak Iban’s collective practices and wisdom support ecological sustainability and reinforce indigenous identity and territorial rights. Other research highlights the need for indigenous participation and legal safeguards in forest governance. ^33^^,^^25^ Integrating customary law into conservation governance can enhance environmental protection and boost community involvement. ^24^ These findings confirm that forest governance in Indonesia is contested, driven by overlapping interests, legal pluralism, and power imbalances between the state, corporations, and indigenous communities. Thus, analyzing power dynamics around forest access rights is key to understanding Indonesia’s current forest governance.

In the Indonesian context, previous research has shown that forest management issues are closely linked to governance, local community livelihoods, and the meaning of natural resources. This situation creates a complex web of power relations; for example, research on edge effects and habitat fragmentation in Mount *Halimun Salak* National Park, ^34^ maintaining local livelihoods ^20^ and the role of forest management in forest management, ^35, 36^ forest resource conflicts, ^37^ which link human pressures to wildlife distribution and the strengthening of local actors’ roles in conservation efforts, ^38^ which provide concrete evidence of how environmental power relations are produced in field practices such as ecological studies and biological inventories in TNGHS which are based on local knowledge and scientific programs (e.g., the IndoBioSys inventory project), ^39^ these dynamics illustrate how conservation narratives, scientific legitimacy, and economic interests construct a competitive arena for access. The case of indigenous communities like *Sungai Utik* also shows a similar pattern. Although the community claims customary rights and practices sustainable management, commercial pressures and national policies shift their position, leading to conflict, local community mobilization, and legal advocacy efforts that lead all elements to form power networks in the struggle for forest access. ^37^ A recent national review of customary governance and the dynamics between the government and the *Dayak Iban Sungai Utik* indigenous community on conservation issues, ^40^ highlights the importance of examining power networks to understand the negotiation process, management dominance, and practices of resistance to such domination.

Various previous studies have shown that unequal power relations arise from multiple factors. In Indonesia, historical records indicated inequality in land access and its manifestation in land ownership. ^41^ However, few studies have examined how marginalized local communities fight for their rights by creating power networks. Imbalances in power distribution and representation of actors within policy networks could hinder the achievement of desired outcomes and progress. ^42^ This study aimed to map the power relations in the struggle for forest access rights by using Mount *Halimun Salak* National Park in West Java and the Sungai Utik Forest in West Kalimantan as cases, with a fair and inclusive forest resource management model.

Furthermore, this study was also relevant within the sociology of government, political ecology, and public administration framework, highlighting the interdisciplinary nature of the research. This multidisciplinary approach was crucial in renewing equitable and inclusive natural resource governance. A deep understanding of the network of power relations at the local level could be the basis for formulating policies that support environmental conservation and respect community rights.

## Methods

### Research approach

This research used a qualitative approach; ^43^ with an interpretive paradigm. This approach was chosen to deeply understand the social realities and networks of power relations between political elites and local communities in the struggle for access to forest resources. The method used is a comparative case study. This research’s comparative case study approach simultaneously considers the macro, meso, and micro dimensions of case-based research. ^44^ This approach emphasizes “cross-location exploration,” namely comparing two locations with different orientations, the Mount *Halimun Salak* National Park, which is more conservation-oriented, and the Sungai Utik forest, which is more industrially oriented. A “case study” is best defined as an intensive study of a single unit that can be generalized to a larger set of units. ^45^ Case studies make it possible to extract lessons from a particular historical experience. ^46^

### Sampling strategy

To elucidate the perceptions of stakeholders (political elites and local communities) regarding the social realities and power relations networks in the struggle for access to forest resources, in-depth interviews with 10 informants in each location, and focus group discussions (FGDs) with representatives of traditional leaders, local communities, government officials, political elites, and environmental activists (NGOs) were conducted. Purposive sampling has been used in research to gain an in-depth understanding of a phenomenon. The interviews were conducted several times (February 2020–September 2020). Meanwhile, the FGD was held on May 15, 2020 in Mount Bogor *Halimun Salak* National Park, and on June 18, 2020 in Sungai Utik Forest. The Ministry of Education, Culture, Research, and Technology of the Republic of Indonesia and the Directorate General of Higher Education, Research, and Technology have approved this research project and determined that this research project meets research ethics standards. Before participating in the research, the participants were fully informed about the objectives and procedures and provided written consent.

### Triangulation

To reduce data bias and increase validity and reality, triangulation of data sources, methods, and perspectives (theories) was carried out to cross-check data and interpretation. ^47– 49^ Triangulation of data sources involves comparing data from various sources using the same technique, through in-depth interviews with various informants, namely community leaders, local communities, NGOs, and government officials. Triangulation of methods was carried out through different data collection methods, namely interviews and FGDs. Triangulation of theory was carried out by using different theories to view the phenomenon of power relations. From the micro side, Foucault’s theory of power relations ^50^ examines how discourse and knowledge shape the behavior of local communities in responding to conflicts over access rights to forest governance with the state. From the macro side, Gramsci’s theory of hegemony ^4^ explains how the structure of power and state domination hegemonizes local communities.

### Participants

We involved five categories of participants representing stakeholders: customary leaders, local communities, government officials, political elites, and environmental activists (NGOs) from the Mount *Halimun Salak* National Park and Sungai Utik Forest areas. These included customary leaders with in-depth knowledge of the history of customary forests. Local communities that have long lived near forest areas (especially those involved in forest conflicts or management). Government officials from the Forestry Service, national park managers, and village/sub-district governments. Local political elites, namely actors involved in forestry policymaking, and environmental activists or NGOs involved in advocating for community rights to forest resources.

### Data collection methods

We conducted in-depth interviews with government officials, village/sub-district governments, political elites, and environmental activists (NGOs). Before conducting the in-depth interviews, ^51^ we identified the informants who would participate in the research. A purposive sampling method was used in data collection, particularly in identifying government officials, village/sub-district governments, political elites, and environmental activists (NGOs). In addition to in-depth interviews, other informants from traditional leaders and local communities were also interviewed through focus group discussions (FGDs), ^51^ as informants were not always available due to their busy schedules in the fields.

Our procedure for interviewing local communities involved granting research permission to the traditional leaders. With this approval, the conventional leaders recommended that residents participate in the interviews. Technically, we interviewed residents in one village on a rotating basis. We also conducted the same interviews with government officials, village/sub-district governments, political elites, and environmental activists (NGOs). Interviews were conducted offline over two months. Before completing the interviews, we made appointments to schedule interviews with the residents. Interviews were recorded with a digital voice recorder and lasted between 1 and 1 hour and 30 minutes. Interviews took place in residents’ homes. The interview guide was structured into several topics, with general, open-ended, non-leading questions and several follow-up questions.

We conducted the FGDs offline at the homes of traditional leaders in each location. These FGDs were conducted for the community only. We sent letters of permission or approval to participate in the research to the traditional leaders in each location. After obtaining approval, we sent discussion materials to each participant to discuss during the FGD. The FGDs lasted three hours. Each FGD participant provided opinions and input on the research topic related to social realities and power relations networks in the struggle to access forest resources. We documented the activities through photographs, participant attendance lists, and FGD minutes, which will be used for analysis in the next stage.

### Data collection instruments and technologies

The following questions guided the interviews:

a. What does the forest mean?
b. Is there legitimacy for forest ownership by the state/community?
c. Are there conflicts in forest management?
d. When did these conflicts begin, and what were their causes?
e. What forms did these conflicts take?
f. Who was involved in the contestation over forest resource management, and what rationales underlie each party’s actions?
g. What was the role of each stakeholder in the struggle for forest resources?
h. Is there local knowledge about forest management?
i. Who holds control over land and forest resources?
j. On what basis is ownership defined?
k. What rights do communities currently have over forest resources?
l. What were the forms and processes of power relations in forest resource management?
m. What are the positions of communities and the state in forest resource management following government forest policies?
n. How was the conflict resolution process for forest management disputes? Who plays a role, and what were their roles?
o. How did communities develop power networks to gain access to forest governance?

Some questions posed to local communities were the same as those posed to stakeholders (government officials, political elites, and NGOs). In addition to interviews, modified data collection was conducted through focus group discussions (FGDs). Discussion topics posed to FGD participants focused on the research objective: mapping the power networks involved in the struggle for access rights to forest resources. Furthermore, the contributions and roles of each stakeholder in forest resource management were also discussed. Meanwhile, for government officials and political elites, discussion topics related to their involvement in providing forest resource management services, including their opinions on the power networks involved in the struggle for access rights to forest resources.

The FGD Guidelines specifically address the following:

a. Forms and processes of power relations.
b. Causes of conflict.
c. Discourse Clash.
d. Power Asymmetry.
e. Resistance and Adaptation.
f. Object of Contestation.

### Data analysis

Data analysis aimed to describe and map the power networks in the struggle for access rights to forest resources. Interviews were transcribed and analyzed through the following stages: (a) Transcription and organization of interview and observation data; (b) Open coding to identify initial themes; (c) Categorization and arrangement of patterns of relationships between themes (e.g., domination, resistance, negotiation, legal recognition, etc.); and (d) Critical interpretation of the network of power relations based on the socio-political context of each location. We used Braun and Clarke’s (2012) thematic analysis, which is appropriate for identifying, evaluating, and formulating key themes expressed by researchers. ^52^ In this analysis, we combined deductive and inductive procedures. As a starting point for categorization, we referred to the conditions related to cooperative relationships between stakeholders (government officials, village/sub-district governments, political elites, and environmental activists), including the theory of Power Networks, which is in a struggle for access rights to forest resources. This analysis encompasses all participants’ statements and perceptions regarding the power networks involved in the struggle for access rights to forest resources. These statements were identified based on perspective themes, which were then concentrated, summarized, coded, and categorized into units of analysis.

### Ethical consideration

This research was approved by the Ethics Committee of the Institute for Research and Community Service, Djuanda University (Number for Ethical Consideration: 001/LPPM/KEP/II/2020) on February 5, 2020. After adequate consultation, the researcher provided written consent to all informants in Mount Halimun Salak National Park and Sungai Utik Forest (Number for Consent Forms from Respondents/Informants: 001/IC/LPPM/II/2020). The informants gave their consent without coercion from anyone. Furthermore, to protect the rights and privacy of respondents, all data obtained will be kept confidential.

## Results

The data from the research results, interviews, and FGDs were processed and analyzed thematically. The results of the analysis can be seen in Table 1. Table 1 explains the stages of thematic analysis using Braun and Clarke’s (2012) concept. Data analysis was carried out through the following stages: (a) Familiarization with Data; (b) Creating Codes; (c) Finding Initial Themes; (d) Review and Develop Themes; (e) Determine and Name the Theme; (f ) Writing a Report.

**Table 1.** Thematic analysis steps according to Braun and Clarke 2012.

Steps	Activity	Output
Familiarization with Data	Reading and understanding the data	Thoroughly Keywords (32 keywords) a. Ancestral heritage b. Life source c. Spirituality d. Valuable assets e. *Dayak Iban* identity f. Customary legitimacy g. State legitimacy h. Dualism of legitimacy i. Conflict with companies j. Conflict with the state k. Physical l. Latent m. Roots of conflict n. Indigenous peoples o. State/central government p. Local government q. Companies r. NGOs s. Customary zoning system t. Customary rules u. Customary sanctions v. Customary control (de facto) w. State control (de jure) x. Dualism of power y. Management and use rights z. Protection and preservation rights aa. Spiritual rights bb. Formal limited rights cc. Collective resistance dd. NGO advocacy ee. Recognition Country ff. Traditional Rituals
Creating Codes	Marking sections of data that have significant meaning	Initial code (12 codes) a. Dominance b. Forest management c. Collective resistance d. Negotiation e. Legal recognition f. Relationship networks g. Customary zoning systems h. Resistance i. Adaptation j. Contestation k. Power relations l. Participatory maps
Finding Initial Themes	Group related codes into potential themes	Subthemes (6 subthemes) a. Forms and processes of power relations b. Causes of conflict c. Color clashes d. Power asymmetry e. Resistance and adaptation f. Objects of contestation
Review and Develop Themes	Ensure that the themes identified truly represent the data	3 sub-themes 1. Community Rights 2. Conflict Resolution 3. Power Relations
Defining and Naming a Theme	Provide a clear label for each theme	2 themes a. Forests as Objects of Contestation b. Power Networks is in a struggle for access rights to forest resources
Writing a Report	Organizing the analysis results into a clear and compelling narrative	Results and discussion

### Description

The concepts of “dualism of legitimacy” and “dualism of power” in this article arise from two sources of authority in forest management: the state and indigenous communities. These concepts are essential to understanding the roots of conflict and power relations in the GHSNP and Sungai Utik cases. Dualism of legitimacy refers to two systems that coexist but do not fully acknowledge each other: state legitimacy (legal-formal) and customary legitimacy (socio-cultural). Dualism of power results from this, as two parallel centers of authority regulate and control forest resources. The state holds de jure power, formal authority backed by law and institutions. Indigenous communities hold de facto power, exercised through local control, customary rules, zoning, and daily forest management.

Based on the analysis results, two themes were obtained in this study: (a) Forest as an object of contestation; (b) Power Networks are in a struggle for access rights to forest resources.

### Forests as objects of contestation

In the Indonesian context, forests are not merely ecological areas but also political spaces fraught with interests and power. In two research locations, Mount *Halimun Salak* National Park (TNGHS) in West Java and the Sungai Utik Customary Forest in West Kalimantan, forests have become arenas of contestation between state actors, political elites, corporate permit holders, and local communities. This contestation involved struggles over access, claims to resource ownership, and the legitimacy of conservation, development, and customary rights narratives.

Mount *Halimun Salak* National Park (TNGHS) is a strategic conservation area on the island of Java, serving as a habitat for numerous endemic species and a vital resource for indigenous communities such as the *Kasepuhan.* Since the national park expansion policy was enacted based on Decree 175/2003, the park’s area has been expanded from 40,000 hectares to 113,357 hectares. ^36^ The expansion of this national park has transformed the community-managed forest area into a national park area, which has resulted in the loss of local community ( *Kasepuhan* indigenous community) access rights to the area, due to conservation policies that tend to be centralistic and oriented solely towards ecological protection. Conflicts arose when traditional community practices carried out for generations are considered contrary to modern conservation principles, without considering local community livelihoods and socio-cultural aspects. Forests became objects of contestation, where the state, through national parks, frames forests as ecological assets that local communities must protect from encroachment. In contrast, local communities saw them as cultural heritage that was part of their identity and life system, including a source of livelihood. The state used conservation discourse to legitimize exclusionary policies, while communities used narratives of customary rights and local wisdom to fight for access to forest management.

Unlike the TNGHS, the Sungai Utik Forest, inhabited by the *Dayak Iban* indigenous people, has received formal recognition as a customary forest. The *Dayak Iban* community was widely known for its success in protecting its forest from encroachment and exploitation by companies holding timber forest product utilization permits (TFPUP). The discourse was that the state grants companies a permit for timber forest utilization (industrial forest), while the community maintains the forest’s sustainability. There are zones designated for conservation and utilization. The conservation issue by indigenous communities against the discourse of industrial forests by the state has resulted in the indigenous community gaining support from various parties. Although the Sungai Utik Forest was recognized as a customary forest, it did not necessarily eliminate the potential for contestation, given that the TFPUP decree has never been revoked. Therefore, the potential for conflict remains, albeit latent. In some cases, local governments have attempted to offer economic cooperation in customary areas with promises of development, which the indigenous community has met with caution. The forest in Sungai Utik was not only a financial or environmental resource, but also a symbol of identity and spirituality.

### Power networks is in a struggle for access rights to forest resources

Several concepts apply in Indonesia when understanding control over land and natural resources. Some land rights were defined as property rights (land rights), while others were defined solely as access rights. Furthermore, the state defined some property and access rights, while others were based on local cultural customs. These differing definitions of rights between the state and indigenous communities led to overlapping recognition of rights by the state and the community. Forestry policies on the conservation and use of timber and other forest products are often directly linked to the traditional rights of communities. It is a fact that local communities often live around the forest and claim control over the forest. However, this community faces a different reality. Legal forest tenure documents do not support traditional community forest ownership. It has undermined the ordinary standing of the community, especially in the face of state interest in the forest. This study demonstrated that all issues related to conflicts between the state and local communities over forest resource management rights stem from conflicts of interest in forest resource management.

In the case of TNGHS, multiple stakeholders have an interest in forests. The primary stakeholders of most significant interest are the local and state communities represented by the TNGHS. Possessing these resources is necessary, especially when each party’s interests need to be considered appropriately. The community values the forest for protection and as a source of livelihood. These concerns conflict with the state’s desire to transform the TNGHS into a protected forest. Both claimed control over all natural resources and the right to control everything from above. This force wants to construct a whole in which, teleologically, everything fits and finds its place. The ministry is based on a division of labor, favoring a few people with social skills and the ability to manage production tools. The TNGHS shows that the state has higher authority while the *Kasepuhan* community is subordinate. These superior and subordinate positions are evident when states seek to exercise power by enforcing laws against those who allegedly violate their rights. This power also includes imprisonment for violations of national park regulations, such as deforestation. However, the antlers were discovered on their farmland, which happened to be in the TNGHS area.

The community’s struggle to obtain access rights to the forest resources, which we refer to as the social-environmental movement, shows the superiority of an environmental management system by the community that is more just for the interests of people’s welfare and environmental sustainability. However, the political and economic elite will always win compared to the lower classes in the political arena in developing countries. Knowledge of the environment will determine the direction of social and environmental movements. In this context, actors fighting for natural resources are divided into three regimes. (1) Organic regime, namely local community knowledge in the form of local wisdom that has lived and managed its territory for centuries; (2) Capitalist regime, namely knowledge reproduced by capitalists in carrying out development and providing employment for local communities; (3) Technological regime, i.e., knowledge reproduced by researchers about biodiversity coming to an area to search for potential plants to be used in commercial applications. ^53^ The three actors with different characters and cultural bases produce three distinct regimes of knowledge about the environment. However, these three bits of knowledge can approach and unite each other, resulting in a common understanding of the environment. In the case of Mount *Halimun Salak* National Park, the battle of the three regimes in managing and utilizing natural resources forms a particular socio-ecological system. ^37^

In the *Kasepuhan* case, the state promoted the power of scientific knowledge by introducing national policies, which directly and indirectly weakened local community knowledge and forced changes in local community attitudes, behaviors, and actions toward forests. It was found. States and local communities may compete and resist to gain power over forest management and use. This conflict created a balance of power. In the case of the TNGHS, the national policy of expanding national parks could only be partially implemented based on the techno-nature concept espoused by the TNGHS. It was hindered by the local community’s knowledge of organic nature ( *Kasepuhan* customs). Most political ecology concepts assume that nature has causal relationships or “agents” that must be considered to explain social and socioecological phenomena better. ^54– 56^

Although there was no overt resistance from local communities, *Kasepuhan’s* power over forest resources was the reason for the limited implementation of the national park’s conservation concept, which was designed based on scientific knowledge. This limited implementation was evidenced by the fact that there was a negotiation process between local communities and TNGHS centers. However, in this case, although the negotiations were still ongoing, more technicalities were already in play, so the state had already won. Nevertheless, disputes remain regarding borders, access, and zoning. In the case of *Sungai Utik*, although formally the *Dayak Iban* community of *Sungai Utik* does not have property rights according to state concepts, they do have access to the area, including all rights: rights of access, withdrawal, management, exclusion, and alienation. These two cases reflect the diverse forms and processes of power relations between the two locations. Despite their different socio-cultural backgrounds, contestation over the forest exhibits similar patterns in TNGHS and *Sungai Utik.* Table 2 provides more details.

**Table 2.** Forms and processes of power relations in TNGH and Sungai Utik Forest.

No	Indicators	TNGHS	*Sungai Utik*
1	Forms and processes of power relations.	The dominance of state and corporate actors using legal legitimacy to control people's living spaces.	Forms of resistance and social transformation have also emerged, championed by local communities to defend their rights and identity.
2	Causes of conflict.	Top-down policies that failed to accommodate local wisdom led to resistance from indigenous communities.	Recognition of customary systems and active community participation in forest management have created a sustainable and inclusive conservation model.
3	Discourse Clash.	The state used the language of conservation and development, while communities used the narrative of customary rights and traditional sustainability.	The state used its right to utilize forests, while communities used the narrative of customary rights with conservation issues.
4	Power Asymmetry.	The state and elites possessed greater legal, regulatory, and economic resources, while communities rely on social networks, local knowledge, and advocacy.	The state and elites possessed greater legal, regulatory, and economic resources, but communities utilize national and international networks to support customary forest management.
5	Resistance and Adaptation.	Local communities were not entirely passive; they actively engaged in overt resistance (demonstrations, lawsuits) and symbolic resistance (traditional rituals, historical narratives).	Local communities actively seek support from various national and international parties through community-based conservation forest campaigns.
6	Object of Contestation.	State conservation interests versus economic exploitation, and the rights of indigenous peoples.	State Industrial Forests versus Indigenous Peoples' Conservation Forests.

## Discussion

### Forests as an object of contestation

Forests were a natural resource, frequently the subject of conflict involving various parties. A frequently raised question regarding natural resources was who has the right to own these resources. To answer this question, Bromley argued that natural resource ownership was divided into four regimes: open access, state ownership, private ownership, and communal ownership. The government had absolute power to determine who had the right to access these resources, and these government policies often created conflicts of interest between its role as an agent of development and a protector of the community. ^57^ The level of conflict varies from covert to overt. The severity of the conflict is influenced by how threatened the ownership rights to the contested natural resources are. Several situations could lead to overt conflict.

First, government-community conflict occurs when the government protected natural resource areas. Using conservation policy instruments, the government determined an area to be a conservation or protected area, as in the case of Mount *Halimun Salak* National Park. This regulation indirectly eliminated the community’s rights to access and conducted activities in the area. Communities lose their rights to regulate, process, and utilize forest resources. This condition caused a conflict of interest between the government and the community. ^37^ State-led and -supported extractive policies eliminate the safety valve for a stable future by eliminating the relationship between society and nature. ^58^

Second, the conflict between the government and society occurred when the government acted as a development agent. With its authority, the government involved the private sector in using natural resources. In contrast, these natural resources were de facto controlled and claimed as communal property of indigenous peoples, for example, in the case of the Sungai Utik Forest conflict in West Kalimantan. Sungai Utik Forest was claimed as the customary forest of the *Dayak Iban Sungai Utik* community. ^37^

Third, the management of natural resources in the era of modernism ignored local wisdom due to the dominance of science, even though local insight is produced by local communities. Modernization began with marginalizing traditional governance based on local knowledge and techniques. This condition has caused local traditions to be uprooted and pushed into the technocratic world. The state intervened to limit access for various reasons, such as two sides of the coin, as if community-based management, even though the state had greater control, ignores local wisdom.

Government intervention in socio-territorial conflicts, accountability mechanisms, and state re-territorialization were paradoxically interconnected in such a way that they hinder the collaborative features and natural resource management capabilities ostensibly supported by community-based approaches. ^59^ Political and legal changes were mediated by power relations, cultural norms, and economic incentives, highlighting that the double-edged sword of rights could pave the way for greater state control over future benefits derived from community territories classified as forest lands. ^10^

Forest destruction in the TNGHS was suspected to be related to the power constellation of natural resource (forest) management. ^60^ A more straightforward definition was needed concerning natural resource management. Principal questions such as: who had the right to manage natural resources? Did the community have room to gain access to natural resource management? The answer to this question was related to the theory of property rights, which was the object of study in ecological politics. Types of property rights were (1) Access rights, namely rights to be able to enter natural resource areas and to take non-extractive rights from the areas; (2) Withdrawal rights, namely rights to produce or utilize natural resources in the area; (3) Management rights, namely the rights to regulate, manage and make rules for the utilization of these natural resources; (4) The right of exclusion, namely the right to determine who is allowed to enter the area, how to access it and how to transfer said access right to another party; (5) The right of transfer, namely the right owned to be able to sell or rent part or all of it to another party. ^61^

Several factors have been identified as the leading causes of conflicts over forest resources. Safitri points out that the political construction and administrative legalization (territorial politics) of natural resources in space, known as space and forests, cause various conflicts. ^62^ Conflict as the complexity of power relations between states and non-state actors, including competition for control of access to natural resources, especially forests. Conflict was a reaction to repressive strategies and mechanisms that have led communities to develop resistance strategies and tactics. ^62^

According to the opinions above, conflicts over forest resources are caused by political structures and administrative legitimacy (territorial politics), conflicts over control of access to natural resources, and strategies of resistance to repressive state measures. It is said that Conflicts in the management and use of forest resources arise because of competing interests, overlapping interpretations of rights and access by states and local communities, and differing underlying knowledge of the parties to the conflict. In the case of TNGHS, introducing new knowledge led to state hegemony over local communities. It changed the community’s knowledge of technical institutions by replacing them with modern knowledge and institutions.

*Kasepuhan* communities no longer have rights and various access to natural resources. Even the battle of knowledge at the bargaining table undermines the functioning of traditional institutions. Community knowledge becomes a subordinate body of knowledge. On the other hand, national knowledge based on technology and capitalism has been prioritized. In this context, Foucault’s approach can be used to see how the collective power of forest management and utilization played a role, where knowledge was seen as power. ^50^ Foucault emphasized the micropolitics of power. Through this micropolitics, the state incorporated local community knowledge with new knowledge and showed how state knowledge can solve forest-related problems through state institutions and various forms of communication.

If there had been cooperation between communities and the government, as in the case of Jambi, this forest resource conflict would not have occurred. The success of the community-government collaboration process will result in the effective use of natural forest resources, educating communities on traditional forest use and conservation of typical forest environments, and the daily use of forest areas in Jambi to develop ecotourism. ^63^ However, the role of the government in achieving ecological governance is more dominant. ^64 ^–^ 65^

### Power networks in the struggle for access rights to forest resources

Based on the phenomena demonstrated by the *Kasepuhan* and *Dayak Iban* Communities of *Sungai Utik*, one impact of conflict at the group level was resilience. Conflict causes indigenous communities to develop resilience. Resilience is defined as the ability of people to recover quickly from shock, injury, etc., and their natural resilience helps them overcome the crisis (Oxford Dictionary). In that case, both the *Kasepuhan* and *Dayak Iban* Communities possessed resilience.

**Figure 1.**
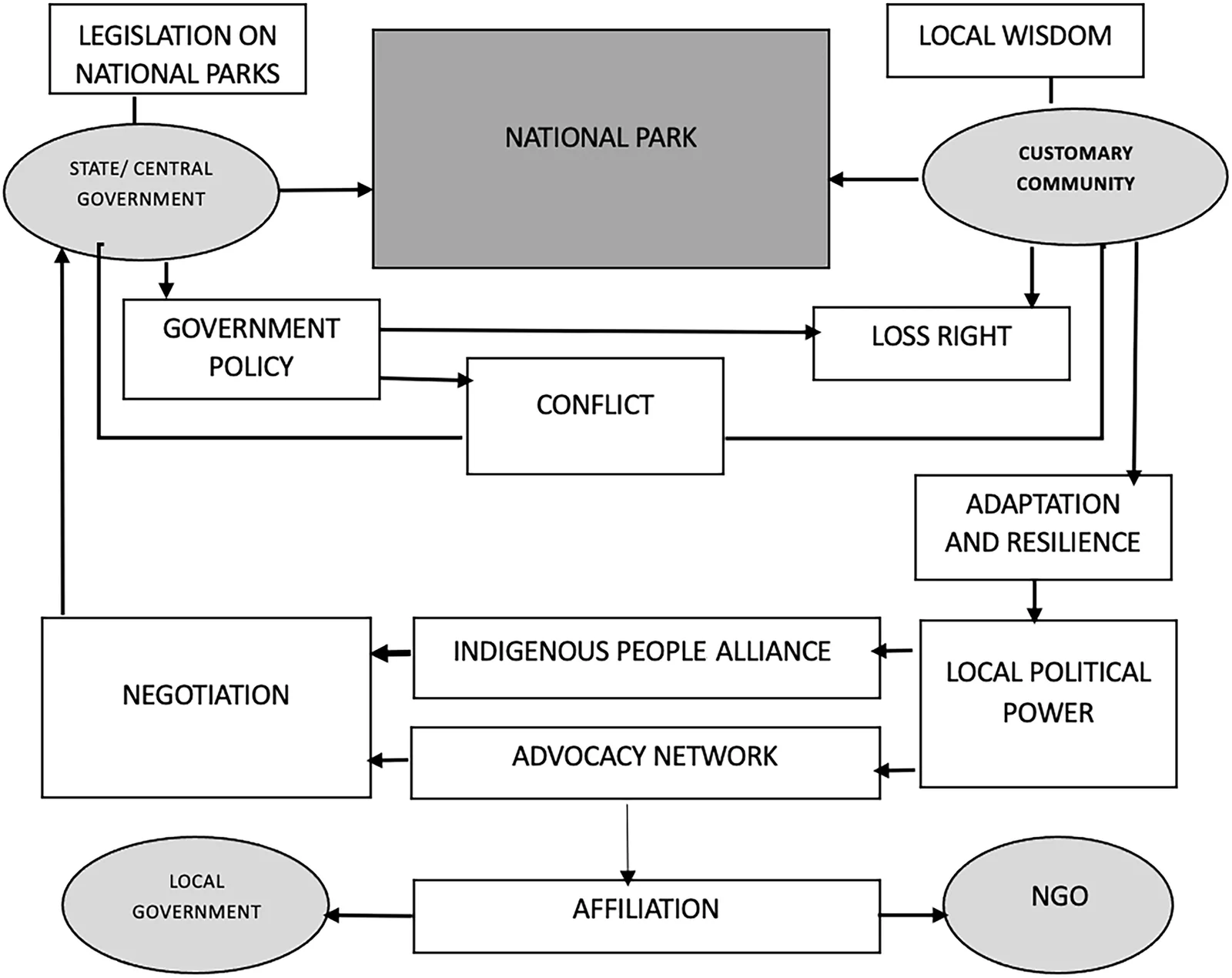
The web of power model of the *Kasepuhan* community.

In the case of the TNGHS, the power network model in Mount *Halimun Salak* National Park can be seen in Figure 1. Figure 1 described that when state institutions dominated local institutions through state policies on national park expansion and cripple customary institutions, this situation has forced customary institutions to change. On the one hand, customary institutions could not benefit from the forest due to a lack of access. However, the community’s ability to adapt to new circumstances has increased due to the conflict with the TNGHS. This ability was more accurately described as resilience. The resilience of the *Kasepuhan* community was demonstrated by their ability to avoid attacks by avoiding open conflict and secretly continuing to cultivate the *leuweung garapan* (cultivated forest) area while still fighting for access rights to the forest. They could also consolidate by forging alliances with other *Kasepuhan* community groups and forming indigenous community alliances, the ability to buy time by negotiating, re-dialoging about boundaries, access, and even zoning, and the ability to embrace other parties by developing their web of power by embracing not only NGOs but also the local government. When access to forest resources became limited, and demands for change from their community increased, the *Kasepuhan* community began to develop other abilities to provide access to the *Kasepuhan* in the local political space, through affiliations they built with local governments or national political elites. In the *Kasepuhan* community, conflict caused change, and this change was evidence that the *Kasepuhan* institution had resilience by developing a web of power whose target was political authority at the local level.

Following the completion of the research, researchers continued to discuss the progress of the community’s negotiations. In their fight for forest rights, the *Kasepuhan* community formed crucial partnerships with local elites and the regional government, demonstrating the potential for collaborative solutions. Constitutional Court Decision No. 35 of 2012 concerning Customary Forests as State Forests raised hopes for the community to gain recognition through regional government regulations. After twenty years of struggle, in 2024, the Sukabumi Regency Government issued Regulation No. 7 of 2024 concerning the Recognition and Protection of Indigenous Communities. This regulation offered hope for the legitimacy of the *Kasepuhan* community, including its customary territory. Through this regional regulation, the community is allowed to regain access rights. However, as of this writing, the boundaries of their customary territory have not yet been determined. This means the community still has to fight to obtain full access rights, even without ownership rights. This situation implies hegemony ^4^ within the *Kasepuhan* community.

The synthesis of Table 2 and Figure 1 shows that the web of power in TNGHS follows a hierarchical, state-centered pattern, in which the state is the dominant actor controlling access to forest resources. This form of power relations is characterized by state and elite dominance through formal legal legitimacy, particularly through national park expansion policies that transform community-managed areas into conservation areas. Within this structure, a strong power asymmetry exists: the state possesses legal, regulatory, and coercive resources, while the Kasepuhan indigenous community is subordinated, relying on social capital, local knowledge, and advocacy networks. According to Table 2, the conflict in TNGHS was triggered by top-down policies that failed to accommodate local wisdom, leading to a clash between the state’s conservation narrative and the community’s customary rights narrative. Figure 1 shows that the community’s power network is not centralized but develops in response to state domination through strategies of resistance and adaptation. Communities engage not only in overt resistance, such as demonstrations or advocacy, but also in covert resistance, such as continuing to manage the land covertly. Furthermore, the web of power in TNGHS shows that indigenous communities build networks with NGOs, local governments, and local political actors to improve their bargaining position. However, these networks are defensive and negotiative, not transformational. This results in limited access, rather than full recognition of community rights. Thus, the main characteristics of the web of power in TNGHS are: (1) state dominance, (2) unequal power relations, (3) adaptive community resistance, and (4) outcomes in the form of limited compromise in the form of access negotiations.

**Figure 2.**
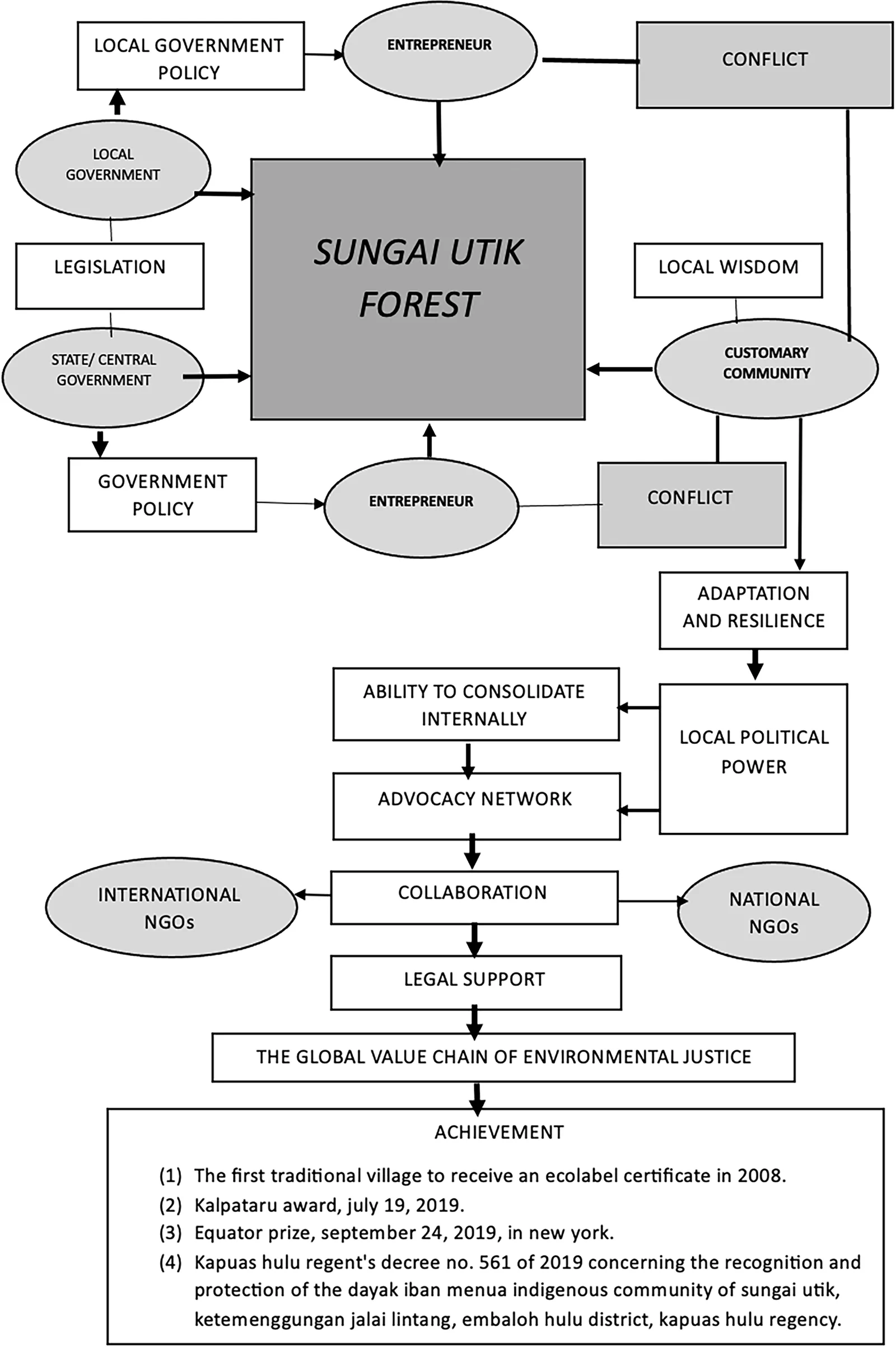
The web of power model of the *Dayak Iban Sungai Utik* community.

The *Dayak Iban* community, distinct from the *Kasepuhan* community, employed unique strategies in conflict resolution. Rather than avoiding attacks, they resisted, expelling entrepreneurs from the area, confiscating their heavy equipment, and demonstrating resilience in defending the area. In their struggle against the state, the *Dayak Iban* community had honed its ability to consolidate internally and with other *Dayak Iban* communities within the *Jalai Lintang Ketemenggungan* (The *Jalai Lintang* clan). They had also expanded their web of power by collaborating with non-governmental organizations (NGOs). The power network in *Sungai Utik* Forest can be seen in Figure 2.

Figure 2 described that forest resource conflicts became increasingly complex when local governments entered the conflict arena with their regional autonomy. In the case of the TNGHS, the local government assisted the *Kasepuhan* community in resolving the conflict with the BTNGHS by supporting the existence of indigenous communities in the TNGHS area. However, in the case of the *Sungai Utik* Forest, the local government exacerbated the conflict by issuing a palm oil plantation permit to a company on the same land. It was a manifestation of a clientelist system, where entrepreneurs, as local elites, exploit their close ties with the state to reproduce power, often at the expense of state expropriation. ^66^ This situation highlights the weakness of state institutions, which have the functional effect of reproducing elites who serve the interests of extractive capital. However, this system was fraught with deep contradictions, leading to conflict and violence that repeatedly endanger these interests. ^66^

In addition to local community actors, the state (Central Government), the state (Regional Government), and business actors, there are also NGO actors. In the case of the TNGHS (National Forest Conservation Area), some NGOs support the BTNGHS program. In contrast, others support the *Kasepuhan* Community’s struggle to obtain access rights to manage the forest. NGOs are central to power relations between both parties in the contest. The *Kasepuhan* Community’s network with certain NGOs is formed due to a shared ideology (defending indigenous peoples) and a shared fate among fellow “indigenous peoples” who generally share the same problems related to tenurial conflicts with the state. In the case of the *Sungai Utik* Forest, the presence of NGOs supports the struggle of the *Sungai Utik Dayak Iban* Community to gain recognition of their customary forest rights. The *Sungai Utik* Community’s struggle is voiced mainly by NGOs claiming to represent and act on behalf of the community. This support from national and international NGOs has influenced the shift in local knowledge of the *Dayak Iban Sungai Utik* Community. These NGOs try to enlighten the community about forest knowledge. NGOs led the community to understand carbon trading, REDD, and demonstrative activity (DA). There was a shift in discourse from the fight for “property rights” to shifting towards global benefits for all parties, the community, the country, and the world.

Furthermore, an important achievement of NGO assistance to the *Dayak Iban* Community of *Sungai Utik* was facilitating the indigenous community to obtain an ecolabeling certificate, namely an award from the Indonesian ecolabel institution with Certificate Number 08/SCBFM/005 given for forest management by the *Sungai Utik* Panjae Menua house (forest management unit of the *Sungai Utik Panjae Menua* house), within the scope of the “sustainable community-based forest management (SCBFM) unit with an area of 9,453.40 hectares”. This ecolabeling certificate proves that community-based forest management ensures social sustainability and conservation. Community-based forest governance creates conditions for achieving better conservation and sustainable use of forest ecosystems, as well as social welfare and poverty reduction for the community. ^59^ These and many other socio-economic needs are part of the global value chain of environmental justice debates. ^67^

In addition to eco-labeling, the results of power relations with various parties have produced the following achievements:

a. Kalpataru award, July 19, 2019.
b. Equator prize, September 24, 2019, in new york.

Kapuas Hulu Regent’s Decree No. 561 Of 2019 Concerning The Recognition and Protection of The *Dayak Iban Menua* Indigenous Community of *Sungai Utik,* The clan of *Jalai Lintang, Embaloh Hulu* District, *Kapuas Hulu* Regency.

Since 2020, the Ministry of Environment and Forestry, through Decree Number 3238/MENLHK-PSKL/PKTHA/PSL.1/5/2020 of 2020, has designated the Menua Sungai Utik Customary Forest, which belongs to the Menua Sungai Utik Dayak Iban indigenous community in Jalai Lintang District, with an area of approximately 9,480 hectares in Kapuas Hulu Regency, West Kalimantan. On July 19, 2023, the Sungai Utik Dayak Iban community received the Gulbenkian Award in Portugal. This achievement further strengthens the Sungai Utik Dayak Iban community’s position in securing access rights to the Sungai Utik forest.

Although the Sungai Utik customary forest received formal recognition, governance remains complex, primarily because the IUPHHK (Forest Product Utilization Permit), a state-issued concession to companies, remains unresolved within the same territory. Recent reforms under the Omnibus Law on Job Creation have restructured licensing and governance systems across Indonesia, centralizing and simplifying natural resource licensing. ^27^^,^^28^ Despite these changes, overlapping claims between newly recognized customary rights and pre-existing company concessions (IUPHHK permits) continue to create legal and administrative uncertainty for all parties involved. This reflects a persistent conflict in Indonesia’s plural legal system between state-based resource control and indigenous rights. ^31^ Inclusive and sustainable forest governance requires coordination between IUPHHK permit holders and forest communities. ^68^ However, overlapping permits set competing interests, customary communities and corporate concessionaires, against each other, and unequal power often disrupts collaboration and forest access management. These governance tensions also appear in protected and conservation forests, where indigenous groups still struggle for full recognition and meaningful participation. ^33^^,^^25^

These cases illustrate governance weaknesses in Indonesia’s resource management, particularly in interactions among regulators, corporate concessionaires, and indigenous communities. Company resistance and weak regulatory oversight often fuel tensions between state policies and realities on the ground, as companies and indigenous rights holders pursue different goals. ^69^ Indonesia’s licensing framework must ensure fairness, transparency, and accountability among all parties. ^70^ Additional studies highlight that incorporating indigenous knowledge and institutions, rather than focusing solely on bureaucratic or corporate processes, is crucial for environmental sustainability. ^24^^,^^71^

The Dayak Iban *Sungai Utik* community has been fighting for the issuance of a Forest Management Permit (IUPHHK) since 1997 and received an ecolabel certificate in 2008. Thus, it took 11 years to achieve their first success in the fight for forest management rights. The *Dayak Iban* community’s success was supported by its power relations with national and international elites and NGOs.

A synthesis of Table 2 and Figure 2 shows that the web of power in Sungai Utik forms a more egalitarian, open, and multi-level (transnational) network-based pattern. Although the state and corporations initially dominated through the granting of concession permits, the Iban Dayak indigenous community was able to reverse this power configuration by strengthening internal solidarity and expanding external networks. Table 2 shows that the community not only resisted but also developed transformational strategies through indigenous conservation-based campaigns and collaboration with national and international actors. Figure 2 demonstrates that the web of power in Sungai Utik is decentralized and expansive, with indigenous communities at its center, connecting various actors, including NGOs, international institutions, the government, and even global mechanisms such as environmental certification. In this context, power asymmetry persists, but it has been successfully negotiated through mobilizing external support, strengthening the community’s position compared to the case of the TNGHS (National Park of the Highlands).

The discourse clash in Sungai Utik has also transformed. Initially, there was a clash between state exploitation narratives and indigenous rights. Later, the community adopted global conservation narratives, such as sustainability and ecolabeling, to strengthen its legitimacy. This demonstrates the community’s ability to strategically frame the situation and broaden support. The resistance they mounted was proactive and confrontational. Their efforts included evictions from companies and strengthening direct control over the area. As a result, the web of power in Sungai Utik has resulted in stronger recognition of indigenous peoples’ rights, including full access to forest management, although the potential for latent conflict remains, as formal permits have not been fully revoked. Thus, the key characteristics of the web of power in Sungai Utik are: (1) an open and multi-level network of power, (2) the central role of indigenous peoples as key actors, (3) the use of collaborative strategies and global advocacy, and (4) outcomes in the form of strengthening the position and recognition of community rights.

Learning from the two cases above, struggles over access rights were unnecessary when the state was present in community issues. Contrasting human resources with natural resources was unnecessary because, in essence, nature must be able to sustain human life. Likewise, human resources, with all their wisdom, must be able to protect the sustainability of nature. State policies to protect or exploit nature could conflict with humans themselves, namely, local communities. Therefore, policies issued by the government must be transparent, participatory, and equitable. Transparency, encouraging equitable policymaking, and ensuring participatory mechanisms were crucial to resolving agrarian conflicts fairly and sustainably. ^72^

The common thread across both cases demonstrates that forests are not merely ecological entities, but also arenas of political contestation involving the state, indigenous communities, corporations, and NGOs. Both cases share similarities in the presence of power imbalances, clashes between conservation and customary rights, and the emergence of community resistance through various strategies, both symbolic and structural. However, there are fundamental differences in the form and outcomes of these power relations. In the TNGHS, power relations are hierarchical with strong state dominance, leading communities to employ more adaptation and negotiation strategies to gain limited access. Meanwhile, in Sungai Utik, power relations are more open and transformative, with indigenous communities able to build cross-scale power networks, including with international NGOs, resulting in stronger recognition of their rights. This difference is also reflected in the power network model, where TNGHS exhibits a more closed, state-centered pattern, while Sungai Utik shows a broader, more flexible network grounded in global solidarity.

The main findings of this study indicate that conflicts over access to forest resources in Mount Halimun Salak National Park (TNGHS) and Sungai Utik have resulted in two distinct webs of power. In TNGHS, the conflict was triggered by conservation-area expansion policies that led to the loss of indigenous peoples’ access to the forest, thereby demonstrating strong state dominance and the subordinate position of the communities. Conversely, in Sungai Utik, the conflict arose from the granting of forest product utilization permits (IUPHHK) in indigenous areas, but power relations developed more egalitarian due to the recognition of indigenous peoples and the support of national and international advocacy networks. These findings contribute significantly to the study of political ecology by emphasizing that power is relational and distributed within a network of actors, not solely monopolized by the state. They also demonstrate how environmental conflicts serve as arenas for the production of knowledge, legitimacy, and resistance. In the study of governance, this study reinforces the importance of an inclusive, collaborative approach and critiques top-down governance models, which have been shown to trigger conflict and injustice in natural resource management.

## Conclusions

Taking the case of the contestation over forest management in Mount *Halimun Salak* National Park and *Sungai Utik* Forest, it was clear that the power relations between local communities and the state were unequal. The state commodifies local knowledge about forest management and other natural resources by introducing new knowledge and development programs (in line with Escobar’s concept of “Knowledge Regimes”). At the intellectual level, state hegemony over local communities occurred (referring to Gramsci’s concept of hegemony). Therefore, contesting natural resources involves three systems: the state, local communities, and corporations, creating a power imbalance where each party fights for rights to forest management (referring to Foucault’s concept of power relations). This contestation gave rise to power networks. The contestation over access rights to forest resources in these two forests produces two different models of power networks. These power networks were formed in response to local communities’ contestation over forest access rights with other parties. Cultural differences, differences in the meaning of forests, and the role of local governments produce different power networks. These different models of power relations also produce different outcomes in conflict resolution. Local communities in Mount *Halimun Salak* National Park reached a point in negotiations that resulted in limited access rights to activities supporting national park programs. Meanwhile, local communities in *Sungai Utik* Forest obtained full access rights, not because the state granted them, but because of legal support obtained through collaboration with various national and international parties.

This model can be generalized and adopted elsewhere, provided that the location has local communities with similar characteristics: local wisdom, the ability to consolidate internally, the ability to build community solidarity that generates social capital, and the presence of NGOs that accompany them in negotiations, alliances, collaborations, advocacy networks, and legal support. Future research can be conducted on collaborative and innovative governance in sustainable forest management.

This study showed that conflicts over forest resource access in the GHSNP and Sungai Utik produce two distinct patterns of power relations: in GHSNP, state dominance is strongly evident due to conservation policies that restrict indigenous peoples’ access, while in Sungai Utik, power relations are more balanced due to the recognition of indigenous peoples and the support of advocacy networks. These findings emphasize that power is relational and distributed within actor networks, and that environmental conflicts serve as a space for the formation of legitimacy, knowledge, and resistance. From a governance perspective, this study emphasizes the importance of an inclusive and collaborative approach while also criticizing top-down models that can give rise to conflict and injustice in natural resource management.

Both cases show that the structure of power networks shapes conflict outcomes. In TNGHS, a state-dominated network led to unequal relations and limited gains. In contrast, Sungai Utik’s open, multi-scale network shifted the power balance and fostered more equitable outcomes for indigenous groups. Ultimately, the core power lies not in the actors alone, but in the capacity to build and manage the power network as a key strategy for securing forest access.

A more concrete policy recommendation for policymakers is to formulate a hybrid forest governance model that balances conservation mandates with recognition of indigenous peoples’ rights. In the context of a policy brief, it should be grounded in a philosophical foundation of ecological justice, a sociological foundation in the form of recognition of sustainable local practices, and a legal basis, such as strengthening the implementation of customary forest recognition. Furthermore, in the form of draft regional regulations, it is necessary to explicitly regulate mechanisms for recognizing indigenous peoples, for determining customary territories through participatory mapping, and for a joint management scheme between the government and local communities.

This study makes a significant contribution to the sociology of governance, political ecology, indigenous peoples’ rights, and forest resource governance.

## Data Availability

This project content the following underlying data: data set of interviews and FGD from the participants. All data underlying the results of this study are available in https://doi.org/10.6084/m9.figshare.30230791 ^36^ Data are available under the terms of the Creative Commons Attribution 4.0 International license (CC-BY 4.0).
