# Supplementary material for: Web of Power in the Struggle for Access Rights to Forest Resources: Comparative Case Studies in Conservation and Industrial Forests
Source: F1000Res. 2026 Aug 17;15:51. Originally published 2026 Jan 13. [Version 3] doi: 10.12688/f1000research.171466.3 (PMC13550930; doi:10.12688/f1000research.171466.3)
Supplement: Peer review history

# Reviewer report: Reviewer response for version 2

**DOI:** 10.5256/f1000research.201389.r492273

**Published:** 2026-07-11

**Authors:** Rozita Arshad

**Affiliations:** 1 Universiti Utara Malaysia, Sintok, Kedah, Malaysia

I have reviewed the revised manuscript based on my previous comments and I am satisfied that the authors have addressed the concerns raised during the review process.

 Based on that improvement by the writer, I am pleased to inform that I have approved the manuscript for indexing. Accordingly, my previous recommendation of “Approved with reservations” may now be updated to “Approved”.

Is the work clearly and accurately presented and does it cite the current literature?

Partly

If applicable, is the statistical analysis and its interpretation appropriate?

Not applicable

Are all the source data underlying the results available to ensure full reproducibility?

Yes

Is the study design appropriate and is the work technically sound?

Yes

Are the conclusions drawn adequately supported by the results?

Yes

Are sufficient details of methods and analysis provided to allow replication by others?

Yes

Reviewer Expertise:

My research area focuses on the intersection of Public Management, Good Governance, and Social Equity, specifically exploring how institutional frameworks and public policies can be designed to empower marginalized groups such as women and persons with disabilities. By integrating expertise in Public Policy and Public Sector Finance, I analyse the "webs of power" within administrative structures to ensure that governance is inclusive, transparent, and conducive to sustainable economic drivers like entrepreneurship. This background aligns directly with the reviewed article, as it provides a robust lens through which to evaluate the struggle for resource access rights and the role of community solidarity in challenging exclusionary state-led management regimes.

I confirm that I have read this submission and believe that I have an appropriate level of expertise to confirm that it is of an acceptable scientific standard.

# Reviewer report: Reviewer response for version 2

**DOI:** 10.5256/f1000research.201389.r496047

**Published:** 2026-07-03

**Authors:** Seunghwan Myeong

**Affiliations:** 1 Inha University, Incheon, Incheon, South Korea

The article investigates forest resource conflicts in Indonesia between state institutions and local/indigenous communities through the theoretical lenses of Foucault’s power relations and Gramsci’s hegemony. It adopts a comparative case study of Mount Halimun Salak National Park (TNGHS) and the Sungai Utik customary forest to examine different “webs of power” in struggles over forest access rights. The findings suggest that top-down conservation policies in TNGHS have contributed to community subordination and restricted access, whereas the Sungai Utik case demonstrates a more egalitarian model shaped by strong community solidarity, legal recognition of customary systems, and broader advocacy networks.

1. **Timely and Relevant Topic:**
  This research is timely and relevant because Indonesia continues to face complex challenges in implementing customary forest rights ( *hutan adat*) and reconciling conservation goals with community access. The study addresses the globally important tension between top-down conservation, sometimes described as “fortress conservation,” and more inclusive models of forest governance. This issue is also relevant to international discussions on biodiversity conservation, indigenous rights, and the 30x30 global conservation agenda. By focusing on “webs of power,” the article offers a useful sociological and political-ecological framework for understanding how marginalized communities navigate state authority, conservation policy, and resource governance.
2. **Comparative Case Study Design:**
  The comparative case study design is appropriate and useful. By comparing the conservation-oriented TNGHS case with the Sungai Utik customary forest case, the study captures different institutional settings, governance regimes, and community strategies. This design enables a cross-location exploration of power relations at the macro, meso, and micro levels. The contrast between a state-dominated conservation model and a more community-based customary forest model provides a meaningful basis for identifying broader lessons about access rights, legal recognition, and community resistance.
3. **Theoretical Grounding:**
  The study is grounded in major social theories, particularly Foucault’s theory of power relations and Gramsci’s theory of hegemony. Foucault’s perspective is useful for examining how discourse, knowledge, and institutional practices shape forest governance and community responses. Gramsci’s concept of hegemony helps explain how state domination is maintained and how counter-hegemonic community strategies emerge. The article’s use of organic, capitalist, and technological regimes provides an additional conceptual structure for analyzing socio-ecological systems in both study locations. However, the theoretical framework would be stronger if the article more clearly explained how these concepts are operationalized in the empirical analysis.
4. **Methodological Rigor:**
  The article employs a qualitative comparative case study approach using purposive sampling, interviews, focus group discussions, observation, and document analysis. The inclusion of ten informants from each location and five categories of stakeholders provides an appropriate basis for qualitative inquiry. The use of thematic analysis based on Braun and Clarke’s framework is also suitable. The presentation of coding stages in Table 1 helps demonstrate the analytical process. However, the article would benefit from more direct interview quotations and clearer explanation of how specific empirical data support each theme. This would strengthen the transparency and credibility of the qualitative findings.
5. **Clarity of Contribution:**
  The article makes a clear contribution to political ecology, forest governance, indigenous rights, and sociology of governance. It addresses a gap in the literature by focusing not only on state control or community resistance, but also on how marginalized communities actively construct networks of power to claim access rights. The identification of two distinct “web of power” models—one more state-dominated and one more egalitarian and solidarity-based—offers a useful conceptual contribution for future comparative research.
6. **Depth of Comparative Analysis:**
  The comparative analysis is one of the article’s main strengths. It contrasts exclusionary, top-down conservation policies in TNGHS with the more inclusive customary forest governance model in Sungai Utik. The article also usefully highlights the clash between state-centered narratives of “techno-nature” and community-centered narratives of “organic nature.” This distinction provides an interesting analytical basis for understanding how different actors define forest value, legitimacy, and authority. Nevertheless, the comparison should be further strengthened by explicitly identifying the key comparative variables, such as legal status, community organization, NGO involvement, state response, and recognition mechanisms.
7. **Policy Implications:**
  The article provides significant policy implications for natural resource governance in Indonesia and beyond. It suggests that forest governance should move away from centralistic and exclusionary conservation models toward more equitable, participatory, and inclusive arrangements. The findings show that legal recognition of customary rights, incorporation of local knowledge, and institutional support for community-based governance are essential for reducing conflict and promoting sustainable forest management. The policy implications would be stronger if the authors provided more concrete recommendations for national park authorities, local governments, NGOs, and policymakers.

**Peer Review Evaluation**

**1. Is the work clearly and accurately presented and does it cite current literature?**

**Answer:** Partly

**Comment:**

 The article is generally clear and presents a strong theoretical foundation using Foucault, Gramsci, political ecology, and access-rights perspectives. However, it only partly satisfies the requirement for current literature. A considerable portion of the references and the data collection period are several years old. Since Indonesian forestry governance, social forestry policy, and customary forest recognition have continued to evolve, the article should incorporate more recent scholarship and policy developments from 2023–2025.

**Constructive Detail:**

**Update Literature:**

 The authors should include recent studies from 2023–2025 on the implementation of Indonesia’s Social Forestry program, customary forest recognition, and the implications of the Job Creation Law/Omnibus Law for forest governance and indigenous land rights.

**Recent Comparative Studies:**

 The authors should integrate recent international literature on inclusive conservation, indigenous-led conservation, and community-based natural resource governance to better position the article within current global debates.

**Policy Context:**

 The article should update the status of the IUPHHK or relevant timber utilization permits in the Sungai Utik case. If the permits remain legally unresolved or have undergone regulatory change, this should be clearly reflected in the analysis.

**2. Is the study design appropriate and is the work technically sound?**

**Answer:** Yes

**Comment:**

 The qualitative comparative case study design is appropriate for examining complex power relations in forest resource governance. Comparing a national park context with a customary/industrial forest context allows the authors to analyze different configurations of state authority, community agency, legal recognition, and advocacy networks. The study is technically sound as a qualitative inquiry, although the empirical presentation should be strengthened with more direct evidence from interviews and FGDs.

**3. Are sufficient details of methods and analysis provided to allow replication by others?**

**Answer:** Yes

**Comment:**

 The article provides sufficient methodological information about case selection, purposive sampling, informant categories, interview questions, focus group discussions, and thematic analysis. Table 1 is useful because it shows the movement from keywords to codes, subthemes, and final themes. This provides a reasonable audit trail for qualitative reproducibility.

 However, the article would be improved by adding more detail on the coding process. The authors should clarify who conducted the coding, whether coding was performed independently or collaboratively, whether there was any validation process, and how disagreements were resolved. These additions would make the analytical process more transparent.

**4. If applicable, is the statistical analysis and its interpretation appropriate?**

**Answer:** Not applicable

**Comment:**

 The study is qualitative and does not rely on statistical testing. Therefore, statistical analysis is not applicable.

**5. Are all the source data underlying the results available to ensure full reproducibility?**

**Answer:** Yes

**Comment:**

 The article provides an adequate basis for qualitative reproducibility through its methodological description and thematic analysis table. Table 1 provides a useful audit trail by showing how raw keywords were transformed into codes, subthemes, and final themes.

 However, because the study involves interviews and FGDs with human participants, the authors should clearly explain how source data were anonymized, what materials were made publicly available, and how confidentiality was protected. This is especially important when dealing with potentially sensitive conflicts involving indigenous communities, state agencies, and forest access rights.

**6. Are the conclusions drawn adequately supported by the results?**

**Answer:** Yes

**Comment:**

 The main conclusion—that community solidarity, legal recognition, and advocacy networks can function as counter-hegemonic forces against exclusionary state-led forest management—is generally supported by the comparative case evidence. The Sungai Utik case provides a persuasive example of how community cohesion and legal recognition can produce a more egalitarian access-rights model. The TNGHS case also supports the argument that top-down conservation policy can subordinate local communities and restrict access.

 Nevertheless, the conclusions would be stronger if the results section included more direct quotations, clearer stakeholder voices, and more explicit links between the empirical evidence and the theoretical interpretation.

**Recommendation**

 To move this article toward a fully approved status, the authors should address the following points:

1. **Literature Update:**
  Integrate at least 5–7 recent references from 2023–2025, especially on Indonesia’s Social Forestry program, customary forest recognition, the Job Creation Law/Omnibus Law, and recent debates on inclusive conservation.
2. **Refine the “Techno-Nature” Concept:**
  Clarify how the concept of “techno-nature” operates in the TNGHS case and how it contrasts with the “organic” regime of community-based forest relations. The theoretical distinction should be made more explicit and analytically robust.
3. **Update Conflict Status:**
  Provide an updated discussion of whether the IUPHHK or related timber utilization permits in the Sungai Utik case have experienced recent legal, administrative, or regulatory changes. This is important to ensure that the analysis of “latent conflict” reflects the current situation.
4. **Strengthen Empirical Evidence:**
  Add direct quotations from interviews and FGDs to support the main claims. The article should show more clearly how community members, customary leaders, government actors, and NGO representatives understand the forest-access conflict.
5. **Clarify the Comparative Framework:**
  Specify the key comparative variables between the two cases, such as forest regime, legal recognition, state authority, community solidarity, NGO support, and outcomes in access-rights struggles.
6. **Improve Theoretical Integration:**
  Foucault, Gramsci, political ecology, and access-rights theory should be more systematically integrated. The article should explain the specific role of each theory in interpreting the empirical findings.
7. **Strengthen Policy Implications:**
  The policy section should provide more concrete recommendations for national park management, local government, indigenous rights recognition, and community-based conservation.The article investigates forest resource conflicts in Indonesia between state institutions and local/indigenous communities through the theoretical lenses of Foucault’s power relations and Gramsci’s hegemony. It adopts a comparative case study of Mount Halimun Salak National Park (TNGHS) and the Sungai Utik customary forest to examine different “webs of power” in struggles over forest access rights. The findings suggest that top-down conservation policies in TNGHS have contributed to community subordination and restricted access, whereas the Sungai Utik case demonstrates a more egalitarian model shaped by strong community solidarity, legal recognition of customary systems, and broader advocacy networks.
8. **Timely and Relevant Topic:**
  This research is timely and relevant because Indonesia continues to face complex challenges in implementing customary forest rights ( *hutan adat*) and reconciling conservation goals with community access. The study addresses the globally important tension between top-down conservation, sometimes described as “fortress conservation,” and more inclusive models of forest governance. This issue is also relevant to international discussions on biodiversity conservation, indigenous rights, and the 30x30 global conservation agenda. By focusing on “webs of power,” the article offers a useful sociological and political-ecological framework for understanding how marginalized communities navigate state authority, conservation policy, and resource governance.
9. **Comparative Case Study Design:**
  The comparative case study design is appropriate and useful. By comparing the conservation-oriented TNGHS case with the Sungai Utik customary forest case, the study captures different institutional settings, governance regimes, and community strategies. This design enables a cross-location exploration of power relations at the macro, meso, and micro levels. The contrast between a state-dominated conservation model and a more community-based customary forest model provides a meaningful basis for identifying broader lessons about access rights, legal recognition, and community resistance.
10. **Theoretical Grounding:**
  The study is grounded in major social theories, particularly Foucault’s theory of power relations and Gramsci’s theory of hegemony. Foucault’s perspective is useful for examining how discourse, knowledge, and institutional practices shape forest governance and community responses. Gramsci’s concept of hegemony helps explain how state domination is maintained and how counter-hegemonic community strategies emerge. The article’s use of organic, capitalist, and technological regimes provides an additional conceptual structure for analyzing socio-ecological systems in both study locations. However, the theoretical framework would be stronger if the article more clearly explained how these concepts are operationalized in the empirical analysis.
11. **Methodological Rigor:**
  The article employs a qualitative comparative case study approach using purposive sampling, interviews, focus group discussions, observation, and document analysis. The inclusion of ten informants from each location and five categories of stakeholders provides an appropriate basis for qualitative inquiry. The use of thematic analysis based on Braun and Clarke’s framework is also suitable. The presentation of coding stages in Table 1 helps demonstrate the analytical process. However, the article would benefit from more direct interview quotations and clearer explanation of how specific empirical data support each theme. This would strengthen the transparency and credibility of the qualitative findings.
12. **Clarity of Contribution:**
  The article makes a clear contribution to political ecology, forest governance, indigenous rights, and sociology of governance. It addresses a gap in the literature by focusing not only on state control or community resistance, but also on how marginalized communities actively construct networks of power to claim access rights. The identification of two distinct “web of power” models—one more state-dominated and one more egalitarian and solidarity-based—offers a useful conceptual contribution for future comparative research.
13. **Depth of Comparative Analysis:**
  The comparative analysis is one of the article’s main strengths. It contrasts exclusionary, top-down conservation policies in TNGHS with the more inclusive customary forest governance model in Sungai Utik. The article also usefully highlights the clash between state-centered narratives of “techno-nature” and community-centered narratives of “organic nature.” This distinction provides an interesting analytical basis for understanding how different actors define forest value, legitimacy, and authority. Nevertheless, the comparison should be further strengthened by explicitly identifying the key comparative variables, such as legal status, community organization, NGO involvement, state response, and recognition mechanisms.
14. **Policy Implications:**
  The article provides significant policy implications for natural resource governance in Indonesia and beyond. It suggests that forest governance should move away from centralistic and exclusionary conservation models toward more equitable, participatory, and inclusive arrangements. The findings show that legal recognition of customary rights, incorporation of local knowledge, and institutional support for community-based governance are essential for reducing conflict and promoting sustainable forest management. The policy implications would be stronger if the authors provided more concrete recommendations for national park authorities, local governments, NGOs, and policymakers.
15. **Peer Review Evaluation**
**1. Is the work clearly and accurately presented and does it cite current literature?**
**Answer:** Partly
**Comment:**
  The article is generally clear and presents a strong theoretical foundation using Foucault, Gramsci, political ecology, and access-rights perspectives. However, it only partly satisfies the requirement for current literature. A considerable portion of the references and the data collection period are several years old. Since Indonesian forestry governance, social forestry policy, and customary forest recognition have continued to evolve, the article should incorporate more recent scholarship and policy developments from 2023–2025.
**Constructive Detail:**
**Update Literature:**
  The authors should include recent studies from 2023–2025 on the implementation of Indonesia’s Social Forestry program, customary forest recognition, and the implications of the Job Creation Law/Omnibus Law for forest governance and indigenous land rights.
**Recent Comparative Studies:**
  The authors should integrate recent international literature on inclusive conservation, indigenous-led conservation, and community-based natural resource governance to better position the article within current global debates.
**Policy Context:**
  The article should update the status of the IUPHHK or relevant timber utilization permits in the Sungai Utik case. If the permits remain legally unresolved or have undergone regulatory change, this should be clearly reflected in the analysis.
**2. Is the study design appropriate and is the work technically sound?**
**Answer:** Yes
**Comment:**
  The qualitative comparative case study design is appropriate for examining complex power relations in forest resource governance. Comparing a national park context with a customary/industrial forest context allows the authors to analyze different configurations of state authority, community agency, legal recognition, and advocacy networks. The study is technically sound as a qualitative inquiry, although the empirical presentation should be strengthened with more direct evidence from interviews and FGDs.
**3. Are sufficient details of methods and analysis provided to allow replication by others?**
**Answer:** Yes
**Comment:**
  The article provides sufficient methodological information about case selection, purposive sampling, informant categories, interview questions, focus group discussions, and thematic analysis. Table 1 is useful because it shows the movement from keywords to codes, subthemes, and final themes. This provides a reasonable audit trail for qualitative reproducibility.
  However, the article would be improved by adding more detail on the coding process. The authors should clarify who conducted the coding, whether coding was performed independently or collaboratively, whether there was any validation process, and how disagreements were resolved. These additions would make the analytical process more transparent.
**4. If applicable, is the statistical analysis and its interpretation appropriate?**
**Answer:** Not applicable
**Comment:**
  The study is qualitative and does not rely on statistical testing. Therefore, statistical analysis is not applicable.
**5. Are all the source data underlying the results available to ensure full reproducibility?**
**Answer:** Yes
**Comment:**
  The article provides an adequate basis for qualitative reproducibility through its methodological description and thematic analysis table. Table 1 provides a useful audit trail by showing how raw keywords were transformed into codes, subthemes, and final themes.
  However, because the study involves interviews and FGDs with human participants, the authors should clearly explain how source data were anonymized, what materials were made publicly available, and how confidentiality was protected. This is especially important when dealing with potentially sensitive conflicts involving indigenous communities, state agencies, and forest access rights.
**6. Are the conclusions drawn adequately supported by the results?**
**Answer:** Yes
**Comment:**
  The main conclusion—that community solidarity, legal recognition, and advocacy networks can function as counter-hegemonic forces against exclusionary state-led forest management—is generally supported by the comparative case evidence. The Sungai Utik case provides a persuasive example of how community cohesion and legal recognition can produce a more egalitarian access-rights model. The TNGHS case also supports the argument that top-down conservation policy can subordinate local communities and restrict access.
  Nevertheless, the conclusions would be stronger if the results section included more direct quotations, clearer stakeholder voices, and more explicit links between the empirical evidence and the theoretical interpretation.
**Recommendation**
  To move this article toward a fully approved status, the authors should address the following points:
16. **Literature Update:**
  Integrate at least 5–7 recent references from 2023–2025, especially on Indonesia’s Social Forestry program, customary forest recognition, the Job Creation Law/Omnibus Law, and recent debates on inclusive conservation.
17. **Refine the “Techno-Nature” Concept:**
  Clarify how the concept of “techno-nature” operates in the TNGHS case and how it contrasts with the “organic” regime of community-based forest relations. The theoretical distinction should be made more explicit and analytically robust.
18. **Update Conflict Status:**
  Provide an updated discussion of whether the IUPHHK or related timber utilization permits in the Sungai Utik case have experienced recent legal, administrative, or regulatory changes. This is important to ensure that the analysis of “latent conflict” reflects the current situation.
19. **Strengthen Empirical Evidence:**
  Add direct quotations from interviews and FGDs to support the main claims. The article should show more clearly how community members, customary leaders, government actors, and NGO representatives understand the forest-access conflict.
20. **Clarify the Comparative Framework:**
  Specify the key comparative variables between the two cases, such as forest regime, legal recognition, state authority, community solidarity, NGO support, and outcomes in access-rights struggles.
21. **Improve Theoretical Integration:**
  Foucault, Gramsci, political ecology, and access-rights theory should be more systematically integrated. The article should explain the specific role of each theory in interpreting the empirical findings.
22. **Strengthen Policy Implications:**
  The policy section should provide more concrete recommendations for national park management, local government, indigenous rights recognition, and community-based conservation.

Is the work clearly and accurately presented and does it cite the current literature?

Partly

If applicable, is the statistical analysis and its interpretation appropriate?

Not applicable

Are all the source data underlying the results available to ensure full reproducibility?

Yes

Is the study design appropriate and is the work technically sound?

Yes

Are the conclusions drawn adequately supported by the results?

Yes

Are sufficient details of methods and analysis provided to allow replication by others?

Yes

Reviewer Expertise:

My research focuses on public administration, digital governance, policy design, institutional analysis, and inclusive governance. I am particularly interested in how public institutions, administrative systems, and policy frameworks shape power relations among the state, communities, and marginalized groups. This background is relevant to the reviewed article because the paper examines how local and indigenous communities construct “webs of power” to challenge exclusionary forest governance and pursue more equitable access to natural resources.

I confirm that I have read this submission and believe that I have an appropriate level of expertise to confirm that it is of an acceptable scientific standard, however I have significant reservations, as outlined above.

# Response

**Published:** 2026-08-11

**Authors:** Rita Rahmawati

**Affiliations:** Public Administration, Universitas Djuanda, Bogor, West Java, Indonesia

**RESPONSE TO THE REVIEWER 3:**

**1. **Thank you for the comment about Timely and Relevant Topic.

 2. Thank you for the comment about Comparative Case Study Design.

 3. Thank you for the comment about Theoretical Grounding.

 Regarding your suggestion about “the theoretical framework, it would be stronger if the article explained more clearly how these concepts are operationalized in the empirical analysis” **, explained in page 7 line 30 to 45, and page 8 line 1-2.**

**"**Foucault’s theory of power relations ^50 ^examines how discourse and knowledge shape the behavior of local communities in responding to conflicts over access rights to forest governance with the state. is operationalized by examining how discourse, knowledge, and everyday institutional practices shape local communities’ perceptions, behaviors, and responses to conflicts over forest access and governance, including how communities accept, negotiate, reinterpret, or resist dominant state narratives and regulations. From the macro side, Gramsci’s theory of hegemony ^4 ^explains how the structure of power and state domination hegemonizes local communities. is used to analyze how state policies, legal frameworks, institutional arrangements, and dominant development or conservation narratives establish and legitimize state authority over forest governance through a combination of coercion and social consent.

 Empirically, these perspectives are examined through interviews, focus group discussions, observations, and policy-document analysis, with a focus on processes of discourse formation, institutional domination, consent, negotiation, resistance, and counter-hegemonic practices. Together, Foucault and Gramsci provide complementary lenses for explaining how macro-level structures of domination are reproduced through micro-level practices and interactions, while also revealing opportunities for local communities to negotiate or challenge existing power relations".

 4. Thank you for the comment about Methodological Rigor.

 Regarding your suggestion about “the article would benefit from more direct interview quotations and clearer explanation of how specific empirical data support each theme. This would strengthen the transparency and credibility of the qualitative findings”, **explained in page 13 line 32 to 38, and page 14, line 8 to 19.**

 "As stated by Informant:

 “From the state’s perspective, the forest belongs to the state. But for us as Indigenous people, the forest is not merely land or a natural resource. It is a legacy from our ancestors—a customary right that has existed since time immemorial and been passed down through generations. Therefore, we feel we have both the right and the responsibility to protect the forest together, ensuring it remains available for use and can be passed on to our children and grandchildren.” (Informant 3, April 14, 2020)".

 As stated by informant:

 “According to our customary law, the question of forest ownership is actually clear-cut. Since the time our ancestors first erected the pillars of our longhouses, this land and the surrounding forest have belonged to us—the people who live here. Ownership has been passed down from parents to children and on to grandchildren. This forest cannot simply be sold or transferred at will; it is not merely a matter of land, but an integral part of our identity as an indigenous community. Thus, under our customary law, this forest belongs to the indigenous people of Sungai Utik. However, we also understand that the state has its own regulations, and we respect them. For this reason, we continue to comply with state rules and recognition. What matters most to us is that the status of our indigenous community and our rights to this forest remain clearly defined and respected under Indonesian law.” (Informant 1, March 7, 2020)

 5. Thank you for the comment about Clarity of Contribution.

 6. Thank you for the comment about Depth of Comparative Analysis.

 Regarding your suggestion about “strengthened by explicitly identifying the key comparative variables, such as legal status, community organization, NGO involvement, state response, and recognition mechanisms”, **We have added the table 2 in page 15, line 5 to 9 and page 16.**

 Tabel 2. The Comparison of Policies Regarding the Recognition and Protection of Customary Law Community Rights in the Mount Halimun Salak National Park Area and the Sungai Utik Forest.

 7. Thank you for the comment about Policy Implications. Regarding your suggestion about “provided more concrete recommendations for national park authorities, local governments, NGOs, and policymakers”, **has deleted former paragraph in page 27, line 13 to 21, and replaced with new explanation in page 27, Line 22 to 32.**

**"**A concrete policy approach is to establish a hybrid forest governance model that balances conservation mandates with legally recognized indigenous rights. Forest management authorities should implement co-management agreements, participatory mapping, and integration of indigenous ecological knowledge into conservation plans. Local governments should strengthen procedures for recognizing indigenous communities and customary territories and resolving overlapping claims. NGOs should support legal literacy, institutional capacity, and community participation, while national policymakers should harmonize conservation, forestry, spatial-planning, and investment regulations and accelerate customary forest recognition. Grounded in ecological justice, this model positions indigenous communities as rights holders and legitimate environmental stewards within a shared governance framework".

 Response to **Peer Review Evaluation:**

**1. **Recent studies are discussed **on page 5,** integrating international literature, literature concerning Indonesia, and the implications of implementing the Omnibus Law.

 Regarding the updating of IUPHHK status, the explanation can be found on **page 24, lines 22 to 24;**

 "Current information indicates that the IUPHHK concession for natural forest utilization in the Sungai Utik area, issued in 2004, was valid until 2024".

**2. **Thank you for positive comment about the study design appropriate and the work technically sound;

 3. Thank you for positive comment about sufficient details of methods and analysis provided to allow replication by others;

 4. This research is qualitative research, no applicable to use statistical analysis;

 5. Thank you for positive comment about all the source data underlying the results available to ensure full reproducibility;

 6. Thank you for positive comment about the conclusions drawn adequately supported by the results;

 Response to **Recommendation**

 To move this article toward a fully approved status, the authors should address the following points:

 1. Thank you for positive comment about literature update. Recent studies are discussed on page 5, integrating international literature, literature concerning Indonesia, and the implications of implementing the Omnibus Law.

 2. Thank you for your suggestion about refine the “Techno-Nature. We have add the explanations on page 16 line 37 to 49 and page 17, line 1 to 4.

 "The concepts of "techno-nature" and "organic nature" draw upon Arturo Escobar’s perspective, which views nature not as a neutral, singular entity separate from society, but rather as something constructed and animated through social, cultural, knowledge-based, and power relations. Differing ways of understanding nature can represent divergent ontologies—specifically, the contrast between the modern (state) ontology that separates nature from culture and the relational ontology of local communities, which views humans and nature as part of a single web of life. In the context of TNGHS, techno-nature can be understood as nature that is constructed, categorized, and governed through the state’s technocratic apparatus. A forest becomes a "national park" not merely due to its ecological characteristics, but through institutional processes such as mapping, boundary setting, zoning, biodiversity inventorying, conservation regulations, permitting, surveillance, and the production of scientific knowledge. Thus, techno-nature operates through mechanisms of scientific knowledge. Conversely, the organic regime governing community-based forest relations, particularly among the Kasepuhan, stems from living, historical connections linking humans, the forest, customary traditions, and the community. Here, the forest is not primarily understood as an administrative object requiring categorization, but rather as an integral part of the community's lived space".

 3. Thank you for suggestions. Regarding the updating of IUPHHK status, the explanation can be found on page 24, lines 22 to 24.

 "Current information indicates that the IUPHHK concession for natural forest utilization in the Sungai Utik area, issued in 2004, was valid until 2024".

 4. Thank you for suggestion, we have added the Explanation in page 13 line 32 to 38, and page 14, line 8 to 19. 

 "As stated by Informant:

 “From the state’s perspective, the forest belongs to the state. But for us as Indigenous people, the forest is not merely land or a natural resource. It is a legacy from our ancestors—a customary right that has existed since time immemorial and been passed down through generations. Therefore, we feel we have both the right and the responsibility to protect the forest together, ensuring it remains available for use and can be passed on to our children and grandchildren.” (Informant 3, April 14, 2020)".

 As stated by informant:

 “According to our customary law, the question of forest ownership is actually clear-cut. Since the time our ancestors first erected the pillars of our longhouses, this land and the surrounding forest have belonged to us—the people who live here. Ownership has been passed down from parents to children and on to grandchildren. This forest cannot simply be sold or transferred at will; it is not merely a matter of land, but an integral part of our identity as an indigenous community. Thus, under our customary law, this forest belongs to the indigenous people of Sungai Utik. However, we also understand that the state has its own regulations, and we respect them. For this reason, we continue to comply with state rules and recognition. What matters most to us is that the status of our indigenous community and our rights to this forest remain clearly defined and respected under Indonesian law.” (Informant 1, March 7, 2020)"

 We have also added the result of FGD in page 14, line 20 to 46, and page 15, line 1 to 4.

 "Regarding the causes of the conflict in Sungai Utik, Informant 8B explained during a focus group discussion (FGD) that the conflict between the indigenous community and the company is rooted in state policies concerning forest utilization permits:

 “The trigger for the conflict is not actually a direct clash between the indigenous community and the state, but rather involves the company that obtained a state permit to utilize the forest. Thus, state policy regarding forest utilization permits is a primary source of the conflict.”

 This explanation was reinforced by Informant 7B, who highlighted the impact of permit issuance on the condition of the forest and the customary rules observed by the community:

 “This conflict stems from state policy on forest utilization. The state grants permits to companies to enter and extract forest products, including logging trees for timber. Yet, the community already possesses customary rules for preserving and protecting the forest.”

 Meanwhile, Informant 1B explained that the conflict does not always manifest as open confrontation; its intensity fluctuates depending on the situation facing the community:

 “At times, the conflict can escalate into open confrontation, but there are also periods when it remains latent. From the outside, it might appear that there are no issues, yet the conflict persists.”

 This perspective was complemented by Informant 10B, who emphasized the historical dimension and the indigenous community's rights over the forest territory:

 “For the community, the forest is not merely a place to extract resources. We believe the forest is a right inherited from our ancestors; therefore, it must be preserved and protected.”

 The informants' accounts indicate that the conflict in Sungai Utik is not solely about forest resource utilization but also involves a contest over authority, management legitimacy, and the meaning attributed to the forest. On one hand, state policy grants companies’ formal legitimacy to utilize the forest; on the other, the indigenous community holds customary rules and legitimacy governing forest protection and use based on ancestral heritage. These differing sources of legitimacy underpin the conflict between interests in conservation and investment versus the rights and community-based forest governance of the indigenous people".

 5. Thank you for suggestion about clarify the comparative framework. We have added the table 2 in page 15, line 5 to 9 and page 16.

 For further details regarding forests as objects of contestation, see Table 2.

 Tabel 2. The Comparison of Policies Regarding the Recognition and Protection of Customary Law Community Rights in the Mount Halimun Salak National Park Area and the Sungai Utik Forest

 6. Thank you for suggestion about improve theoretical integration, we have added explanation in Page 19, Line 3 to 20.

 "To analyze forest conflict and governance in TNGHS and Sungai Utik, the theories of Foucault and Gramsci, along with political ecology and access theory, can be integrated into a unified analytical framework. Foucault’s work explains how the state produces knowledge about forests through classification, mapping, zoning, regulation, and licensing, thereby transforming the forest into an object that can be governed; Gramsci’s concepts explain how such knowledge and policies gain legitimacy through hegemony—manifested in conservation discourses within TNGHS and in development and utilization practices in Sungai Utik; the political ecology perspective reveals how these power relations generate conflict and inequality regarding the distribution of authority, benefits, and management burdens; meanwhile, access theory clarifies that formal rights or legality do not equate to the actual ability to derive benefits from the forest, as access is also shaped by capital, technology, knowledge, social networks, and institutional power. In TNGHS, the contestation primarily pits the state’s conservation regime against the community's access to their living space. In contrast, in Sungai Utik, the conflict highlights the clash between the legality of corporate forest utilization and the community's customary legitimacy in preserving the forest as an ancestral heritage. Thus, both cases demonstrate that forest conflict is not merely a matter of resource access but also a contestation of knowledge, legitimacy, power, and authority in determining how forests are defined and managed".

 7. Thank you for suggestion about strengthen policy implications. We have deleted former paragraph in page 27, line 13 to 21, and replaced with new explanation in page 27, Line 22 to 32.

 "A concrete policy approach is to establish a hybrid forest governance model that balances conservation mandates with legally recognized indigenous rights. Forest management authorities should implement co-management agreements, participatory mapping, and integration of indigenous ecological knowledge into conservation plans. Local governments should strengthen procedures for recognizing indigenous communities and customary territories and resolving overlapping claims. NGOs should support legal literacy, institutional capacity, and community participation, while national policymakers should harmonize conservation, forestry, spatial-planning, and investment regulations and accelerate customary forest recognition. Grounded in ecological justice, this model positions indigenous communities as rights holders and legitimate environmental stewards within a shared governance framework".

# Reviewer report: Reviewer response for version 1

**DOI:** 10.5256/f1000research.189076.r450352

**Published:** 2026-05-05

**Authors:** Rozita Arshad

**Affiliations:** 1 Universiti Utara Malaysia, Sintok, Kedah, Malaysia

**Article Summary**

 The study investigates forest resource conflicts in Indonesia between the state and local communities through the lens of Foucault’s power relations and Gramsci’s hegemony theories. It uses a comparative case study of Mount Halimun Salak National Park (TNGHS) and the Sungai Utik customary forest to map different "webs of power". The results show that top-down conservation policies in TNGHS led to community subordination and loss of access, while Sungai Utik achieved a more egalitarian model through strong community solidarity and legal recognition of customary systems.

**1. Timely and Relevant Topic:** This research is highly timely as Indonesia continues to grapple with the legal implementation of customary forest rights ( *hutan adat*) following significant judicial and legislative shifts. The study addresses the globally relevant tension between "fortress conservation" (top-down exclusion) and "inclusive governance," making it pertinent for international readers interested in the 30x30 global conservation targets. By focusing on "webs of power," it provides a contemporary sociological framework for understanding how marginalized communities navigate the "Social Forestry" era in Indonesia. Such an approach is particularly relevant for international policymakers, researchers, and practitioners seeking balanced strategies for achieving global conservation objectives while minimizing conflicts with local and indigenous communities.

**2. Comparative Case Study Design**: The research effectively employs a comparative case study method to analyze different forest management orientations: the conservation-oriented TNGHS and the industrially oriented Sungai Utik. This design is a significant strength, as it allows for "cross-location exploration" that simultaneously considers macro, meso, and micro dimensions of power. The use of two distinct sites provides a robust basis for generating generalized lessons from specific historical experiences.

**3. Theoretical Grounding**: The study is strongly grounded in established social theories through a sophisticated triangulation of analytical perspectives. At the micro level, Michel Foucault’s theory of power relations is employed to examine how discourse, knowledge, and institutional practices shape the behavior and responses of local communities, while at the macro level, Antonio Gramsci’s theory of hegemony is applied to explain forms of state domination and their influence on local socio-political structures. The integration of these theoretical perspectives within the Organic, Capitalist, and Technological regimes offers a comprehensive framework for analyzing the socio-ecological systems across both study locations.

**3. Methodological Rigor: **The study employs a rigorous and transparent methodological approach using purposive sampling involving ten informants from each location representing five categories of stakeholders, including customary leaders, government representatives, non-governmental organizations, and community actors. To enhance the validity and reliability of the findings, the study applies triangulation of both data sources, including interviews and focus group discussions (FGDs), and theoretical perspectives. The data were systematically analyzed using thematic analysis based on the six-stage framework developed by Braun and Clarke (2012), which was clearly operationalized and presented in Table 1. Furthermore, the study demonstrates strong ethical compliance through formal research approval procedures and the acquisition of written informed consent from all informants.

**4. Clarity of Contribution: **The article makes a clear contribution to the fields of political ecology and sociology of governance. It explicitly addresses a gap in existing literature by focusing on how marginalized communities actively fight for their rights by creating power networks. The identification of two distinct "web of power" models (one state-dominated and one egalitarian/solidarity-based) provides a valuable conceptual tool for future research.

**5. Depth of Comparative Analysis: **The comparative analysis is detailed and insightful, as summarized in Table 2. It contrasts the "top-down" exclusionary policies in TNGHS with the "inclusive conservation model" in Sungai Utik . The analysis goes beyond simple comparison by examining the "discourse clash" between state narratives of "techno-nature" and community narratives of "organic nature" .

**6. Policy Implications: **The study offers significant policy implications for natural resource governance in Indonesia. It advocates for a transition from centralistic, exclusionary conservation to more "equitable and inclusive" models. The findings suggest that legal recognition of customary rights and the incorporation of local knowledge are essential for reducing conflict and ensuring sustainable forest management.

**Peer Review Evaluation**

**1. Is the work clearly and accurately presented and does it cite current literature?**

**Answer: Partly**

**Comment**: While the article is clearly presented and provides a strong theoretical foundation using Foucault and Gramsci, it only partly fulfills the requirement for citing current literature. A significant portion of the references and the data collection period (2020) are several years old. To be considered fully current in the context of Indonesian forestry policy and global political ecology studies, the authors should incorporate more recent scholarship and policy updates from the 2023–2025 period.

**Constructive Detail: **

1. Update Literature: The authors should include recent studies (2023–2025) on the implementation of Indonesia's Omnibus Law (Job Creation Law) and its specific impacts on customary forest recognition and national park management.
2. Recent Comparative Studies: Integrate 2024–2025 research regarding global trends in "inclusive natural resource governance" to better contextualize the findings within the current international academic discourse.
3. Policy Context: Update the status of the IUPHHK (timber utilization permits) mentioned in the Sungai Utik case to reflect any regulatory changes or revocations.

**2. Is the study design appropriate and is the work technically sound?**

**Answer: Yes**

**Comment** The qualitative comparative case study design is highly appropriate for mapping the complexities of power. Comparing a National Park with an industrially managed forest allows for a robust analysis of different power configurations.

**3. Are sufficient details of methods and analysis provided to allow replication?**

**Answer: Yes**

**Comment:** The methodology is transparent, detailing purposive sampling, the list of informants, and the specific interview guiding questions. The thematic analysis stages are well-defined.

**4. If applicable, is the statistical analysis and its interpretation appropriate?**

**Answer: Not applicable**

**Comment:** The study is qualitative; no statistical testing was required.

**5. Are all the source data underlying the results available to ensure full reproducibility?**

**Answer: Yes**

**Comment:** Table 1 provides an excellent audit trail of the thematic analysis, showing the transformation of raw keywords from the raw data into final themes. This provides sufficient transparency for qualitative reproducibility.

**6. Are the conclusions drawn adequately supported by the results?**

**Answer: Yes**

**Comment:** The conclusion that community solidarity acts as a "counter-hegemony" to state power is well-supported by the Sungai Utik case study evidence.

**Recommendation:**

 To transition this article to a fully "Approved" status, the following points must be addressed:

1. Literature Update (Critical): Integrate at least 5–7 references from 2023–2025. These should specifically focus on the recent evolution of Indonesia’s "Social Forestry" program and the impact of the Job Creation Law (Omnibus Law **)** on customary land rights.
2. Refine "Techno-nature" Concept: Clarify how this framework specifically interacts with the "Organic" regime in TNGHS to ensure theoretical robustness. 
3. Conflict Status Update: Provide an update on whether the IUPHHK permits in Sungai Utik have faced any recent legal changes to ensure the "latent conflict" analysis reflects the 2026 context.

Is the work clearly and accurately presented and does it cite the current literature?

Partly

If applicable, is the statistical analysis and its interpretation appropriate?

Not applicable

Are all the source data underlying the results available to ensure full reproducibility?

Yes

Is the study design appropriate and is the work technically sound?

Yes

Are the conclusions drawn adequately supported by the results?

Yes

Are sufficient details of methods and analysis provided to allow replication by others?

Yes

Reviewer Expertise:

My research area focuses on the intersection of Public Management, Good Governance, and Social Equity, specifically exploring how institutional frameworks and public policies can be designed to empower marginalized groups such as women and persons with disabilities. By integrating expertise in Public Policy and Public Sector Finance, I analyse the "webs of power" within administrative structures to ensure that governance is inclusive, transparent, and conducive to sustainable economic drivers like entrepreneurship. This background aligns directly with the reviewed article, as it provides a robust lens through which to evaluate the struggle for resource access rights and the role of community solidarity in challenging exclusionary state-led management regimes.

I confirm that I have read this submission and believe that I have an appropriate level of expertise to confirm that it is of an acceptable scientific standard, however I have significant reservations, as outlined above.

# Response

**Published:** 2026-05-28

**Authors:** Rita Rahmawati

**Affiliations:** Public Administration, Universitas Djuanda, Bogor, West Java, Indonesia

Thank you for your constructive comments that have helped improve the quality of our manuscript.

 Following your valuable comments, we have revised the manuscript based on your suggestions, adding references to the latest literature and policies from 2023 to 2025. All revisions have been highlighted and discussed point by point. We have implemented your suggestions and resubmitted the revised manuscript. Once again, we appreciate the reviewers' insights and hope this revised manuscript is worthy of your approval for publication.

# Response

**Published:** 2026-07-09

**Authors:** Rita Rahmawati

**Affiliations:** Public Administration, Universitas Djuanda, Bogor, West Java, Indonesia

Response to The Reviewer Comment:

 1. Thank you for your positive comment about timely and relevant topic; Comparative Case Study Design; Theoretical Grounding; Methodological Rigor; Clarity of Contribution; Depth of Comparative Analysis; and Policy Implications.

 Response to the Peer Review Evaluations:

 1. Thank you for your constructive comment. We appreciate your recognition of the article’s theoretical foundation and clear presentation. Several references and our data collection period were outdated and did not reflect recent changes in Indonesian forestry policy and global political ecology research. We have revised the manuscript by: (1) adding literature and policy references from 2023–2025 to strengthen its relevance; (2) updating sections on forest governance, indigenous rights, and political ecology to match current debates and policy developments; and (3) placing our findings in the context of these ongoing discussions. These changes ensure our content is current and aligned with the latest policy and academic discourse.

 (1) Regarding the comment: “Update Literature”: The authors should include recent studies (2023–2025) on the implementation of Indonesia's Omnibus Law (Job Creation Law) and its specific impacts on customary forest recognition and national park management. (Nasional), We have revised the introduction section, page 5 lines 21 to 48, and page 6 lines 1 to 4.

 (2) Regarding the comment: Recent Comparative Studies: Integrate 2024–2025 research regarding global trends in "inclusive natural resource governance" to better contextualize the findings within the current international academic discourse (Internasional global cases), We have revised the introduction section, page 5 lines 1 to 21.

 (3) Regarding the comment: Policy Context: Update the status of the IUPHHK (timber utilization permit) mentioned in the Sungai Utik case to reflect any changes or revocations of the regulation. (IUPHHK Regulation), We have revised the discussion section. We have removed lines 11 to 22 on page 21 and replaced them with an explanation on lines 23 to 44, and on page 22 lines 1 to 8.

 2. Thank you for positive comment about the study design is appropriate and the work technically is sound.

 3. Thank you for the reviewer’s constructive comment about sufficient details of methods and analysis provided to allow replication.

 4. Thank you for your comments. Our research used qualitative methods, so it does not use statistical analysis.

 5. Thank you for your positive comment about all the source data underlying the results available to ensure full reproducibility.

 6. Thank you for your positive comment about the conclusions drawn adequately supported by the results.

 Response to the Recommendation:

 1. Literature Update (Critical): Integrate at least 5–7 references from 2023–2025. These should specifically focus on the recent evolution of Indonesia’s "Social Forestry" program and the impact of the Job Creation Law (Omnibus Law **)** on customary land rights.

**Answer:**

 Recent international studies show that inclusive governance is key to environmentally sustainable and socially just development. Kato and Manchidi (2025) emphasized its role in responsible, equitable supply chain management in Africa [22]. Bibliometric research by Faheem et al. (2023) revealed a global trend toward international collaboration, green innovation, and inclusive natural resource governance [23]. Recent work also highlights the increasing importance of customary institutions and indigenous participation. For instance, integrating customary law into marine protected area management in Indonesia can improve conservation and local legitimacy [24]. Likewise, Swardhana and Jenvitchuwong (2023) argued that indigenous participation in land management is vital to equitable governance and protecting these communities under environmental and development pressures [25].

 These developments show a broader shift in global environmental governance. Previously, models were hierarchical and top-down. Now, there is a move toward adaptive, participatory, and multi-level governance systems. These new systems value local ecological knowledge, customary institutions, and shared decision-making. In many countries, environmental conflicts arise not only from competition over resources but also from struggles for recognition, legitimacy, and authority in governance. Batiran et al. (2023) studied changes in Kelimutu National Park [26]. Their work showed how conservation policies can reshape indigenous institutions, creating new tensions between state agendas and local communities. The cases of Mount Halimun Salak National Park and the Sungai Utik customary forest contribute to global discussions about inclusive governance, political ecology, and indigenous environmental rights.

 2. Refine "Techno-nature" Concept: Clarify how this framework specifically interacts with the "Organic" regime in TNGHS to ensure theoretical robustness. 

**Answer:**

 Recent developments in Indonesia’s forestry governance have been strongly influenced by the implementation of the Omnibus Law on Job Creation (Law No. 11 of 2020), later reinforced through Law No. 6 of 2023. The regulation introduced significant changes to environmental and forestry governance by simplifying licensing systems, centralizing authority, and accelerating investment mechanisms. Although the policy was intended to improve economic efficiency and national competitiveness, several studies have highlighted its implications for environmental governance, particularly concerning indigenous peoples’ rights, customary forest recognition, and conservation management  that the implementation of the Omnibus Law has created challenges for forest decentralization in Indonesia by weakening political and administrative decentralization in forestry governance [27]; [28]; [29]. This condition can affect the recognition of customary forests and limit local communities’ participation in forest management processes. Furthermore, recent reviews of studies on the Omnibus Law have highlighted ongoing controversies surrounding its legal, social, and ecological implications, particularly regarding environmental protection and forest governance [30]. These studies suggest that regulatory simplification under the Omnibus Law may weaken participatory mechanisms and create overlapping authority between conservation policies, investment interests, and indigenous territorial claims. Similar concerns were also raised by Imamulhadi et al. (2025), who argued that the transformation of customary environmental law in Indonesia continues to face significant challenges due to the dominance of state-centered legal frameworks and the limited integration of customary governance systems into formal environmental policies [31].

 Indigenous forest governance in Indonesia is fundamentally shaped by struggles over power and recognition. Studies on the Dayak Iban in West Kalimantan show that customary forest recognition is crucial for sustainable management and local protection [32]. The Dayak Iban’s collective practices and wisdom support ecological sustainability and reinforce indigenous identity and territorial rights. Other research highlights the need for indigenous participation and legal safeguards in forest governance [33]; [25]. Integrating customary law into conservation governance can enhance environmental protection and boost community involvement [24]. These findings confirm that forest governance in Indonesia is contested, driven by overlapping interests, legal pluralism, and power imbalances between the state, corporations, and indigenous communities. Thus, analyzing power dynamics around forest access rights is key to understanding Indonesia’s current forest governance.

 3. Conflict Status Update: Provide an update on whether the IUPHHK permits in Sungai Utik have faced any recent legal changes to ensure the "latent conflict" analysis reflects the 2026 context.

**Answer:**

 Since 2020, the Ministry of Environment and Forestry, through Decree Number 3238/MENLHK-PSKL/PKTHA/PSL.1/5/2020 of 2020, has designated the Menua Sungai Utik Customary Forest, which belongs to the Menua Sungai Utik Dayak Iban indigenous community in Jalai Lintang District, with an area of approximately 9,480 hectares in Kapuas Hulu Regency, West Kalimantan. On July 19, 2023, the Sungai Utik Dayak Iban community received the Gulbenkian Award in Portugal. This achievement further strengthens the Sungai Utik Dayak Iban community's position in securing access rights to the Sungai Utik forest.

 Although the Sungai Utik customary forest received formal recognition, governance remains complex, primarily because the IUPHHK (Forest Product Utilization Permit), a state-issued concession to companies, remains unresolved within the same territory. Recent reforms under the Omnibus Law on Job Creation have restructured licensing and governance systems across Indonesia, centralizing and simplifying natural resource licensing [27]; [28]. Despite these changes, overlapping claims between newly recognized customary rights and pre-existing company concessions (IUPHHK permits) continue to create legal and administrative uncertainty for all parties involved. This reflects a persistent conflict in Indonesia’s plural legal system between state-based resource control and indigenous rights [31]. Inclusive and sustainable forest governance requires coordination between IUPHHK permit holders and forest communities [69]. However, overlapping permits set competing interests, customary communities and corporate concessionaires, against each other, and unequal power often disrupts collaboration and forest access management. This governance tensions also appear in protected and conservation forests, where indigenous groups still struggle for full recognition and meaningful participation [33]; [25].

 These cases illustrate governance weaknesses in Indonesia's resource management, particularly in interactions among regulators, corporate concessionaires, and indigenous communities. Company resistance and weak regulatory oversight often fuel tensions between state policies and realities on the ground, as companies and indigenous rights holders pursue different goals [70]. Indonesia's licensing framework must ensure fairness, transparency, and accountability among all parties [71]. Additional studies highlight that incorporating indigenous knowledge and institutions, rather than focusing solely on bureaucratic or corporate processes, is crucial for environmental sustainability [24]; [72].

# Reviewer report: Reviewer response for version 1

**DOI:** 10.5256/f1000research.189076.r467727

**Published:** 2026-04-09

**Authors:** Harshit Pant

**Affiliations:** 1 G.B.Pant National Institute of Himalayan Environment, Almora, Uttrakhand, India

The paper investigates how power relations shape conflicts over forest access in Indonesia. Using a qualitative comparative case study approach, the authors analyze Mount Halimun Salak National Park (TNGHS) in West Java and the Sungai Utik customary forest in West Kalimantan.

- In **TNGHS**, expansion of the national park (from 40,000 to 113,357 hectares) restricted indigenous Kasepuhan communities’ access, creating conflict between conservation policies and local livelihoods.
- In **Sungai Utik**, conflict stemmed from a state-issued industrial forest permit (IUPHHK) overlapping with customary lands. Despite the permit not being revoked, recognition of customary rights and strong community solidarity fostered more egalitarian power relations.

The study highlights that forests are not only ecological assets but also contested political spaces. Power relations are dynamic, shaped by state legitimacy, customary rights, NGO advocacy, and community resistance. The authors conclude that inclusive governance requires recognizing local knowledge, balancing ecological conservation with socio-cultural needs, and addressing asymmetries in power.

- **Timely and relevant topic**: The paper addresses pressing issues of forest governance, indigenous rights, and conservation conflicts in Indonesia, with broader implications for global environmental governance.
- **Comparative case study design**: Juxtaposing conservation-oriented (TNGHS) and industrial-oriented (Sungai Utik) contexts enriches the analysis and demonstrates the variability of power networks.
- **Theoretical grounding**: The integration of Foucault’s relational power and Gramsci’s hegemony provides a strong conceptual lens for analyzing contestation and resistance.
- **Methodological rigor**: Use of triangulation (data sources, methods, theories) strengthens validity. Thematic analysis is well-structured, with clear coding and theme development.
- **Clarity of contribution**: While the study maps power relations, the paper could more explicitly articulate how its findings advance theory in political ecology or governance studies.
- **Depth of comparative analysis**: The two cases are described well, but the cross-case synthesis could be expanded to highlight similarities and differences more systematically.
- **Policy implications**: The discussion touches on inclusive governance but could provide more concrete recommendations for policymakers, especially regarding balancing conservation mandates with indigenous rights.
- **Literature engagement**: Some references are mentioned broadly (e.g., REDD+ in Vietnam, land grabbing in India), but the connection to the Indonesian cases could be more critically developed.
- Improve flow in the **Introduction**, which currently lists multiple global examples; synthesizing them into thematic clusters would enhance readability.
- Clarify terminology (e.g., “dualism of legitimacy” and “dualism of power”) with consistent definitions.
- Tables and figures could be used more effectively to visualize the web of power networks.

✅ Overall Recommendation

 The article presents a valuable and well-structured study on forest governance conflicts in Indonesia. With revisions to strengthen comparative insights and policy relevance, it has strong potential for indexing after peer review.

Is the work clearly and accurately presented and does it cite the current literature?

Partly

If applicable, is the statistical analysis and its interpretation appropriate?

Partly

Are all the source data underlying the results available to ensure full reproducibility?

Partly

Is the study design appropriate and is the work technically sound?

Partly

Are the conclusions drawn adequately supported by the results?

Partly

Are sufficient details of methods and analysis provided to allow replication by others?

Partly

Reviewer Expertise:

Forest ecology and biodiversity conservation

I confirm that I have read this submission and believe that I have an appropriate level of expertise to confirm that it is of an acceptable scientific standard, however I have significant reservations, as outlined above.

# Response

**Published:** 2026-05-01

**Authors:** Rita Rahmawati

**Affiliations:** Public Administration, Universitas Djuanda, Bogor, West Java, Indonesia

Thank you for your positive comment about:

- Timely and relevant topic,
- Comparative case study design;
- Theoretical grounding;
- Methodological rigor;

Regarding the comment: “Clarity of contribution: While the study maps power relations, the paper could more explicitly articulate how its findings advance theory in political ecology or governance studies,” We have revised the discussion section, page 9, column 2, lines 11 to 33, and the “Conclusions” section, on page 10, column 1, lines 15-31, which  have marked with red writing.

 Regarding the comment about "Depth of comparative analysis: The two cases are described well, but the cross-case synthesis could be expanded to highlight similarities and differences more systematically", I have revised in the “discussion” section on page 9, column 1, on lines 57-66, and on page 9, column 2, on line 1-10, have marked it with green text.

 Regarding the comment about "Policy implications: The discussion touches on inclusive governance but could provide more concrete recommendations for policymakers, especially regarding balancing conservation mandates with indigenous rights", We have corrected it and placed it in the “conclusion” section, page 10 column 1, on rows 51-66, and page 10 column 2, on rows 1-2, and have marked it in blue.

 Regarding comments about "Literature engagement: Some references are mentioned broadly (e.g., REDD+ in Vietnam, land grabbing in India), but the connection to the Indonesian cases could be more critically developed", and suggestions about "Improve flow in the Introduction, which currently lists multiple global examples; synthesizing them into thematic clusters would enhance readability", have been corrected in the introduction section on page 2 column 2, on lines 23-40, and has been marked in brown.

 Regarding the comment about "Clarify terminology (e.g., 'dualism of legitimacy' and 'dualism of power') with consistent definitions", We have added a note on page 17, after table 1. Thematic analysis steps according to Braun and Clarke 2012, I marked them in green.

 Regarding the comment about “Tables and figures could be used more effectively to visualize the web of power networks,” we have corrected the “discussion” section, page 7, column 2, lines 60-66, and page 8, column 1, lines 1-25; we have also added it to page 8, column 2, line 66, and page 9, column 1, lines 1-42. We have also added a complete synthesis to the “conclusion” section on page 10, column 1, lines 32-41. All of these texts are highlighted in purple.

 Thank you for your recommendations regarding our manuscript. Regarding revisions based on the comments, we have conveyed each point in the response system and in the manuscript (in colored text).

# Response

**Published:** 2026-05-28

**Authors:** Rita Rahmawati

**Affiliations:** Public Administration, Universitas Djuanda, Bogor, West Java, Indonesia

Thank you for your helpful comments, suggestions, and careful review of the manuscript. Your feedback improved the clarity, methods, context, and overall quality of our paper.

 In response to your comments, we have revised the manuscript by strengthening the connections between the findings and conclusions, improving the explanation of the thematic analysis process, and incorporating recent literature and policy references from 2023 to 2025. These changes aim to enhance the manuscript's relevance and academic contribution. All revisions have been highlighted and addressed point by point in the response document. We have also resubmitted a revised version of the manuscript based on your suggestions. We appreciate your thoughtful feedback and hope that the revised manuscript addresses all concerns and meets your approval.

# Response

**Published:** 2026-07-09

**Authors:** Rita Rahmawati

**Affiliations:** Public Administration, Universitas Djuanda, Bogor, West Java, Indonesia

Response to the Reviewer Comment:

 1. Thank you for your positive comment about timely and relevant topic; Comparative case study design; Theoretical grounding; Methodological rigor

 2. Regarding the comment: “Clarity of contribution: While the study maps power relations, the paper could more explicitly articulate how its findings advance theory in political ecology or governance studies,”

 We have revised the discussion section, page 23, lines 14 to 28.

 "The main findings of this study indicate that conflicts over access to forest resources in Mount Halimun Salak National Park (TNGHS) and Sungai Utik have resulted in two distinct webs of power. In TNGHS, the conflict was triggered by conservation-area expansion policies that led to the loss of indigenous peoples' access to the forest, thereby demonstrating strong state dominance and the subordinate position of the communities. Conversely, in Sungai Utik, the conflict arose from the granting of forest product utilization permits (IUPHHK) in indigenous areas, but power relations developed more egalitarian due to the recognition of indigenous peoples and the support of national and international advocacy networks. These findings contribute significantly to the study of political ecology by emphasizing that power is relational and distributed within a network of actors, not solely monopolized by the state. They also demonstrate how environmental conflicts serve as arenas for the production of knowledge, legitimacy, and resistance. In the study of governance, this study reinforces the importance of an inclusive, collaborative approach and critiques top-down governance models, which have been shown to trigger conflict and injustice in natural resource management".

 and the “Conclusions” section, on page 24, lines 7-16.

 "This study showed that conflicts over forest resource access in the GHSNP and Sungai Utik produce two distinct patterns of power relations: in GHSNP, state dominance is strongly evident due to conservation policies that restrict indigenous peoples' access, while in Sungai Utik, power relations are more balanced due to the recognition of indigenous peoples and the support of advocacy networks. These findings emphasize that power is relational and distributed within actor networks, and that environmental conflicts serve as a space for the formation of legitimacy, knowledge, and resistance. From a governance perspective, this study emphasizes the importance of an inclusive and collaborative approach while also criticizing top-down models that can give rise to conflict and injustice in natural resource management".

 3. Regarding the comment about "Depth of comparative analysis: The two cases are described well, but the cross-case synthesis could be expanded to highlight similarities and differences more systematically",

 We have revised in the “discussion” section on page 23, on lines 1-13.

 "The common thread across both cases demonstrates that forests are not merely ecological entities, but also arenas of political contestation involving the state, indigenous communities, corporations, and NGOs. Both cases share similarities in the presence of power imbalances, clashes between conservation and customary rights, and the emergence of community resistance through various strategies, both symbolic and structural. However, there are fundamental differences in the form and outcomes of these power relations. In the TNGHS, power relations are hierarchical with strong state dominance, leading communities to employ more adaptation and negotiation strategies to gain limited access. Meanwhile, in Sungai Utik, power relations are more open and transformative, with indigenous communities able to build cross-scale power networks, including with international NGOs, resulting in stronger recognition of their rights. This difference is also reflected in the power network model, where TNGHS exhibits a more closed, state-centered pattern, while Sungai Utik shows a broader, more flexible network grounded in global solidarity".

 4. Regarding the comment about "Policy implications: The discussion touches on inclusive governance but could provide more concrete recommendations for policymakers, especially regarding balancing conservation mandates with indigenous rights",

 We have corrected it and placed it in the “conclusion” section, page 24, on rows 23-31.

 "A more concrete policy recommendation for policymakers is to formulate a hybrid forest governance model that balances conservation mandates with recognition of indigenous peoples' rights. In the context of a policy brief, it should be grounded in a philosophical foundation of ecological justice, a sociological foundation in the form of recognition of sustainable local practices, and a legal basis, such as strengthening the implementation of customary forest recognition. Furthermore, in the form of draft regional regulations, it is necessary to explicitly regulate mechanisms for recognizing indigenous peoples, for determining customary territories through participatory mapping, and for a joint management scheme between the government and local communities".

 5. Regarding comments about "Literature engagement: Some references are mentioned broadly (e.g., REDD+ in Vietnam, land grabbing in India), but the connection to the Indonesian cases could be more critically developed", and suggestions about "Improve flow in the Introduction, which currently lists multiple global examples; synthesizing them into thematic clusters would enhance readability", have been corrected in the introduction section on page 4, on lines 36-47:

 "Various cross-country studies have shown that forest management policies and REDD+ programs are not merely technical instruments. Instead, they are arenas of power relations that determine access to and distribution of resource benefits. A common thread across these cases is the duality of these policies. On one hand, they promote conservation and governance. On the other hand, they can strengthen the control of dominant actors and marginalize local communities, especially when power and participation are not inclusive. This situation is relevant to Indonesia. Despite a relatively strong REDD+ institutional design, regulatory barriers remain  [19]. Unequal benefits persist for smallholders and indigenous communities due to weak political representation [20]. Challenges continue with funding, land boundaries, and community engagement [21]. Strengthening equitable access, participation, and distribution of power is key to ensuring REDD+ is not only ecologically effective but also socially just".

 6. Regarding the comment about "Clarify terminology (e.g., 'dualism of legitimacy' and 'dualism of power') with consistent definitions",

 We have added a note on page 12, after table 1:

 "Description:

 The concepts of "dualism of legitimacy" and "dualism of power" in this article arise from two sources of authority in forest management: the state and indigenous communities. These concepts are essential to understanding the roots of conflict and power relations in the GHSNP and Sungai Utik cases. Dualism of legitimacy refers to two systems that coexist but do not fully acknowledge each other: state legitimacy (legal-formal) and customary legitimacy (socio-cultural). Dualism of power results from this, as two parallel centers of authority regulate and control forest resources. The state holds de jure power, formal authority backed by law and institutions. Indigenous communities hold de facto power, exercised through local control, customary rules, zoning, and daily forest management".

 7. Regarding the comment about “Tables and figures could be used more effectively to visualize the web of power networks,” we have corrected the “discussion” section, page 19, lines 14-34:

 "The synthesis of Table 2 and Figure 1 shows that the web of power in TNGHS follows a hierarchical, state-centered pattern, in which the state is the dominant actor controlling access to forest resources. This form of power relations is characterized by state and elite dominance through formal legal legitimacy, particularly through national park expansion policies that transform community-managed areas into conservation areas. Within this structure, a strong power asymmetry exists: the state possesses legal, regulatory, and coercive resources, while the Kasepuhan indigenous community is subordinated, relying on social capital, local knowledge, and advocacy networks. According to Table 2, the conflict in TNGHS was triggered by top-down policies that failed to accommodate local wisdom, leading to a clash between the state's conservation narrative and the community's customary rights narrative. Figure 1 shows that the community's power network is not centralized but develops in response to state domination through strategies of resistance and adaptation. Communities engage not only in overt resistance, such as demonstrations or advocacy, but also in covert resistance, such as continuing to manage the land covertly. Furthermore, the web of power in TNGHS shows that indigenous communities build networks with NGOs, local governments, and local political actors to improve their bargaining position. However, these networks are defensive and negotiative, not transformational. This results in limited access, rather than full recognition of community rights. Thus, the main characteristics of the web of power in TNGHS are: (1) state dominance, (2) unequal power relations, (3) adaptive community resistance, and (4) outcomes in the form of limited compromise in the form of access negotiations".

**Response to the Peer Review Evaluation**

**1. **We've added relevant literature and improved the flow of the Introduction, including several global examples, Indonesian case studies, and synthesized them. Corrections can be found in the introduction on page 4, lines 36-47:

 "Various cross-country studies have shown that forest management policies and REDD+ programs are not merely technical instruments. Instead, they are arenas of power relations that determine access to and distribution of resource benefits. A common thread across these cases is the duality of these policies. On one hand, they promote conservation and governance. On the other hand, they can strengthen the control of dominant actors and marginalize local communities, especially when power and participation are not inclusive. This situation is relevant to Indonesia. Despite a relatively strong REDD+ institutional design, regulatory barriers remain [19]. Unequal benefits persist for smallholders and indigenous communities due to weak political representation [20]. Challenges continue with funding, land boundaries, and community engagement [21]. Strengthening equitable access, participation, and distribution of power is key to ensuring REDD+ is not only ecologically effective but also socially just".

 2. Thank you for the reviewer’s valuable comment. This study employed a qualitative research design, considered appropriate for exploring the complexity of governance practices and local wisdom within the research context. The study's methodological rigor was strengthened through the triangulation of data sources, methods, and theoretical perspectives. In addition, the thematic analysis was conducted systematically through data coding, categorization, and theme development to ensure consistency and credibility of interpretation. To further improve the technical soundness of the study, we have clarified several methodological procedures in the revised manuscript.

 3. Thank you for your constructive feedback. We recognize the need for clear methodological detail to support transparency and replicability. In response, we revised the Methodology section. We added clear explanations of participant selection, data collection, triangulation techniques, and the stages of thematic analysis, including coding and theme development. These revisions enable other researchers to understand and replicate our methods in similar qualitative contexts.

 4. Thank you for the reviewer’s comment. This study employs a qualitative research approach and therefore does not involve statistical analysis. Instead, the study emphasizes qualitative rigor through triangulation of data sources, methods, and theoretical perspectives, as well as systematic thematic analysis involving coding, categorization, and theme development. To avoid ambiguity, we have clarified in the revised manuscript that the interpretation of findings is based on qualitative analytical procedures rather than statistical methods.

 5. We appreciate the reviewers’ constructive comments. While ethical considerations and participant confidentiality prevent full public disclosure of raw data from this qualitative study, we have detailed our data sources, collection procedures, and analysis in the manuscript to ensure transparency and facilitate reproducibility in similar contexts.

 6. Thank you for your valuable feedback. We recognize the need for our conclusions to be fully supported by our research findings. In response, we revised the Conclusion to directly link the findings to the thematic analysis and to clarify how each conclusion follows from the evidence gathered through interviews, observations, and documents. These changes improve consistency, clarity, and validity.
